# *ZRANB3* is an African-specific type 2 diabetes locus associated with beta-cell mass and insulin response

**DOI:** 10.1038/s41467-019-10967-7

**Published:** 2019-07-19

**Authors:** Adebowale A. Adeyemo, Norann A. Zaghloul, Guanjie Chen, Ayo P. Doumatey, Carmen C. Leitch, Timothy L. Hostelley, Jessica E. Nesmith, Jie Zhou, Amy R. Bentley, Daniel Shriner, Olufemi Fasanmade, Godfrey Okafor, Benjamin Eghan, Kofi Agyenim-Boateng, Settara Chandrasekharappa, Jokotade Adeleye, William Balogun, Samuel Owusu, Albert Amoah, Joseph Acheampong, Thomas Johnson, Johnnie Oli, Clement Adebamowo, South Africa Zulu Type 2 Diabetes Case-Control Study, Ji Chen, Meng Sun, Fraser Pirie, Tommy Carstensen, Cristina Pomilla, Elizabeth H. Young, Manjinder Sandhu, Andrew P. Morris, Inês Barroso, Mark I. McCarthy, Anubha Mahajan, Eleanor Wheeler, Ayesha A. Motala, Francis Collins, Georgia Dunston, Charles N. Rotimi

**Affiliations:** 1 Center for Research on Genomics and Global Health, National Human Genome Research Institute, National Institutes of Health, Bethesda, 20892 MD USA; 2 Division of Endocrinology, Diabetes and Nutrition, Department of Medicine, University of Maryland School of Medicine, Baltimore, 21201 MD USA; 3 Program in Personalized and Genomic Medicine, University of Maryland School of Medicine, Baltimore, 21201 MD USA; 4 University of Lagos, Lagos, Nigeria; 5 University of Nigeria Teaching Hospital, Enugu, Nigeria; 6 University of Science and Technology, Kumasi, Ghana; 7 National Human Genome Research Institute, National Institutes of Health, Bethesda, 20892 MD USA; 8 College of Medicine, University of Ibadan, Ibadan, Nigeria; 9 University of Ghana Medical School, Accra, Ghana; 10 Department of Epidemiology and Public Health; Institute of Human Virology; Greenebaum Comprehensive Cancer Center, School of Medicine, University of Maryland, Baltimore, 21201 MD USA; 11 Oxford NIHR Biomedical Research Centre, Oxford, OX3 7JX UK; 12 National Institutes of Health, Bethesda, 20892 MD USA; 13 National Human Genome Center at Howard University, Washington, 20059 DC USA; 14 Wellcome Sanger Institute, Hinxton, Cambridge, CB10 1SA UK; 15 Wellcome Centre for Human Genetics, University of Oxford, Roosevelt Drive, Oxford, OX3 7BN UK; 16 Department of Diabetes and Endocrinology, University of KwaZulu-Natal, Durban, 4013 South Africa; 17 Department of Medicine, University of Cambridge, Cambridge, CB2 0QQ UK; 18 Department of Biostatistics, University of Liverpool, Liverpool, L69 3GL UK; 19 MRC Epidemiology Unit, University of Cambridge School of Clinical Medicine, Institute of Metabolic Science, Cambridge Biomedical Campus, Cambridge, CB2 0QQ UK; 20 Oxford Centre for Diabetes, Endocrinology and Metabolism, University of Oxford, Oxford, OX3 7LE UK; 21 Oxford NIHR Biomedical Research Centre, Oxford, OX3 7JX UK

**Keywords:** Developing world, CRISPR-Cas9 genome editing, Genome-wide association studies, Type 2 diabetes

## Abstract

Genome analysis of diverse human populations has contributed to the identification of novel genomic loci for diseases of major clinical and public health impact. Here, we report a genome-wide analysis of type 2 diabetes (T2D) in sub-Saharan Africans, an understudied ancestral group. We analyze ~18 million autosomal SNPs in 5,231 individuals from Nigeria, Ghana and Kenya. We identify a previously-unreported genome-wide significant locus: *ZRANB3* (Zinc Finger RANBP2-Type Containing 3, lead SNP *p* = 2.831 × 10^−9^). Knockdown or genomic knockout of the zebrafish ortholog results in reduction in pancreatic β-cell number which we demonstrate to be due to increased apoptosis in islets. siRNA transfection of murine *Zranb3* in *MIN6* β-cells results in impaired insulin secretion in response to high glucose, implicating *Zranb3* in β-cell functional response to high glucose conditions. We also show transferability in our study of 32 established T2D loci. Our findings advance understanding of the genetics of T2D in non-European ancestry populations.

## Abstract (web summary)

Type 2 diabetes (T2D) is prevalent in populations worldwide, however, mostly studied in European and mixed-ancestry populations. Here, the authors perform a genome-wide association study for T2D in over 5,000 sub-Saharan Africans and identify a locus, *ZRANB3*, that is specific for this population.

## Introduction

The genetic architecture of type 2 diabetes (T2D, MIM:125853) in Africa remains largely understudied. While the reduced linkage disequilibrium (LD) characteristic of African populations was used to refine and fine map the original *TCF7L2* genetic association^1,2^, genome-wide and/or high throughput studies of the genetics of T2D in Africa remain limited to a genome-wide linkage analysis^3^, and a large-scale replication study^4^, both from the Africa America Diabetes Mellitus (AADM) Study. African American populations, on the other hand, have been studied more comprehensively including several genome-wide association studies (GWAS) and meta-analysis of GWAS^5^. However, African American populations should not be used as proxies for populations in Africa because of differences in genetic (African Americans have ~20% European admixture) as well as nongenetic risk factors (including lifestyle and behavioral factors). Therefore, despite the advances over the last decade in our understanding of the role of genetic variants influencing T2D risk and the identification of the role of the genes in pathophysiology, data from Africa remains scarce.

In the present study, we conduct a GWAS of T2D in Africa using data from over 5,000 Africans enrolled from Nigeria, Ghana, and Kenya as part of the Africa America Diabetes Mellitus (AADM) study^3,6^, and extend the transferability of previously reported T2D loci in Africa. We identify a novel genome-wide significant locus for T2D—the *Zinc Finger RANBP2-Type Containing 3* (*ZRANB3*) gene. Functional studies of the *ZRANB3* ortholog in zebrafish show that either genomic knockout or antisense knockdown of the gene leads to reduction in β-cell number in the developing embryo which we demonstrate to be due to a reproducible increase in apoptosis in islets. Notably, *Zranb3* knockdown in cultured *MIN6* β-cells results in impaired secretion of insulin in response to high glucose. Our findings represent an advance in our knowledge of the genetics of T2D in sub-Saharan Africa.

## Results

### Characteristics of discovery sample

The characteristics of the 5231 AADM study participants (2342 T2D cases and 2889 controls) are shown in Supplementary Table 1. T2D cases were older than controls (mean age 55 years versus 46 years). Mean body mass index (BMI) was similar between cases and controls. However, cases had a significantly bigger waist circumference than controls (mean 93.7 cm versus 88.5 cm). Fasting glucose values indicate that most of the T2D patients had not achieved glycemic control at the time of enrollment with a median fasting glucose of 153 mg/dl (8.5 mmol/L) and more than three-quarters of the participants having fasting glucose values greater than 109 mg/dl (6.1 mmol/L) at the time of enrollment into the study. PC plots of the genotypes showed clustering of the study participants by geography and ethnolinguistic group (Supplementary Fig. 1).

### Discovery genetic association analysis

**Fig. 1:**
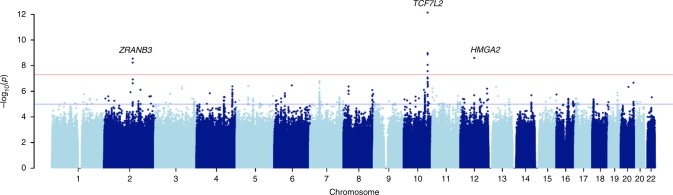
Manhattan plot of discovery GWAS: the AADM study

**Fig. 2:**
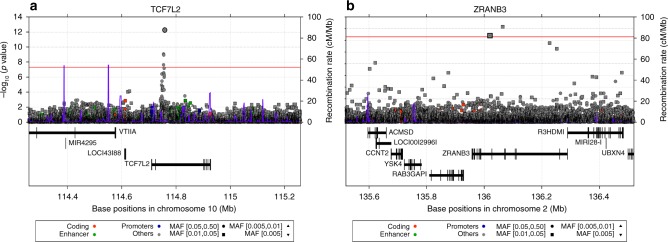
Regional association plots for *TCF7L2* and *ZRANB3* in the AADM GWAS for T2D

The distribution of association statistics for the genome-wide association analysis is shown in the Manhattan plot (Fig. 1). There was minimal inflation of the association statistics (*λ* = 1.013, Supplementary Fig. 2). Analysis with or without BMI in the association model yielded essentially the same findings. Three genome-wide significant loci were identified (Table 1): *TCF7L2* (lead single-nucleotide polymorphism (SNP) rs7903146, T allele frequency = 0.331, *p* = 7.288 × 10^−13^, score test), *HMGA2* (lead marker rs138066904, deletion frequency = 0.096, *p* = 2.516 × 10^−9^, score test) and *ZRANB3*—Zinc Finger RANBP2-Type Containing 3 (lead SNP chr2:136064024, T allele frequency = 0.034, *p* = 2.831 × 10^−9^, score test). *TCF7L2* is an established T2D risk locus and the lead SNP of *TCF7L2* (rs7903146) in the present study is the same lead SNP reported in most GWAS of T2D to date (Fig. 2). *HMGA2* is also a known T2D locus in both Europeans and African Americans. However, the genome-wide significant *HMGA2* variant in the present study is a deletion (CCTAG/C), not a SNP like other *HMGA2* markers that have previously been found to be genome-wide significant for T2D in Europeans (leading SNP rs2258238, 68.5 kb away from the deletion) and in African Americans (leading SNP rs343092, 38.6 kb away). The LD between the deletion and these other SNPs is low (*r*^2^ 0.052 and 0.003, respectively) in this study of sub-Saharan Africans.

**Table 1:** SNPs most significantly associated with T2D in 5231 sub-Saharan Africans (the AADM study)

CHR	BP	SNP	Gene	NEA	EA	EA freq.	Score statistic	Variance	*P* value
10	114758349	rs7903146	*TCF7L2*	C	T	0.331	146.777	418.603	7.288e−13
10	114754071	rs34872471	*TCF7L2*	T	C	0.391	129.338	449.438	1.055e−09
10	114754784	rs35198068	*TCF7L2*	T	C	0.390	128.338	448.171	1.342e−09
12	66289518	rs138066904	*HMGA2*	CCTAG	C	0.096	72.999	149.997	2.516e−09
2	136064024	2:136064024	*ZRANB3*	A	T	0.036	−39.914	45.135	2.831e−09
2	136019729	rs1465146591	*ZRANB3*	C	A	0.069	−51.974	79.670	5.783e−09
10	114754088	rs7901695	*TCF7L2*	T	C	0.504	125.252	473.671	8.664e−09
10	114756041	rs4506565	*TCF7L2*	A	T	0.502	120.971	473.349	2.695e−08
10	114747860	rs386418874	*TCF7L2*	C	CGT	0.238	96.130	321.980	8.449e−08
10	114754601	rs59326375	*TCF7L2*	G	A	0.223	96.808	330.571	1.012e−07
2	136226838	2:136226838	*ZRANB3*	C	T	0.039	−36.964	48.694	1.176e−07
7	44062728	rs116050569	Intergenic	T	C	0.063	53.858	105.402	1.555e−07
10	114755496	rs4132115	*TCF7L2*	G	T	0.222	94.370	326.884	1.793e−07
7	44071333	rs531496714	*RASA4CP*	C	T	0.067	53.800	106.545	1.866e−07

*EA* effect allele, *NEA* noneffect allele

### Replication and annotation of ZRANB3

The association findings implicate *ZRANB3* as a candidate locus for T2D as it has not been previously reported in relation to T2D. The two genome-wide significant SNPs have a frequency of 3.6% and 6.9%, respectively (Table 2) and the *r*^2^ between them is 0.66. They both appear to be African-specific as they are not present in other populations as evaluated through the 1000 Genomes and gNOMAD databases. For evaluation of replication of ZRANB3 in another African ancestry population, we examined these variants in South African Zulu T2D cases and controls from the Durban Diabetes Case–Control Study (DCC) and the Durban Diabetes Study (DDS)—Table 2. The leading SNPs in *ZRANB3* in AADM each showed consistency of direction of effect in the Zulu GWAS, despite the latter study showing lower effect allele frequencies (chr2:136064024: T allele frequency 0.9% Zulu versus 3.4% AADM; rs1465146591 (chr2:136019729) A allele frequency 2.6% Zulu versus 6.9% in AADM). Although these findings did not reach nominal significance, the combined *p* values for the two leading SNPs across the discovery and replication samples remained genome-wide significant (Table 2).

**Table 2:** Association tests for the *ZRANB3* T2D locus in AADM discovery (*n* = 5231) and Zulu replication (*n* = 2578) studies

Marker	EA/NEA	AADM EAF	AADM β (SE)	AADM *p* value	Zulu EAF	Zulu *β* (SE)	Zulu *p* value	Meta-analysis *Z*	Meta-analysis *p* value
2:136064024	T/A	0.036	−0.884 (0.149)	2.83E−09	0.009	−0.157 (0.133)	2.38E−01	−5.541	3.015E−08
rs1465146591	A/C	0.069	−0.652 (0.112)	5.78E−09	0.026	−0.102 (0.058)	7.74E−02	−5.781	7.446E−09

*EA* effect allele, *NEA* noneffect allele

*ZRANB3* is a protein-coding gene with nucleic acid binding and endonuclease activity. The *ZRANB3* transcript is the target of nonsense-mediated decay (NMD) and is expressed in tissues relevant to T2D, including adipose tissue, skeletal muscle, pancreas, and liver (Supplementary Fig. 3). We identified haplotype blocks around the two genome-wide significant *ZRANB3* SNPs and identified 35 and 43 target genes, respectively, from the significant t*rans*-eQTL-gene associations in each haplotype block using the Framingham Heart Study (FHS) eQTL database (Supplementary Table 2). We also identified five common target genes for *cis*-expression quantitative trait loci (eQTLs) in addition to *ZRANB3*. Overlaying these associations with known T2D loci from the GWAS Catalog highlighted two known-T2D genes associated with *cis*-eQTLs (*MCM6*, *DARS*) and four with *trans*-eQTLs (*DGKB*, *GTF3AP5-AGMO*, *IL23R/IL12RB2*, *SLC44A4*). It is noteworthy that there is a ClinVar record of a duplication in *ZRANB3* associated with gestational diabetes (ClinVar Accession SCV000191187.1: see Web Resources). Also, data from the Rat Genome Database shows that the syntenic region contains QTLs for insulin levels (insulin level quantitative trait locus (QTL) 44) and glucose level (glucose level QTLs 66 and 67) in the rat. As of October 2018, there is no *ZRANB3* variant in the NHGRI-EBI GWAS Catalog or in OMIM

### Replication of known T2D loci

We investigated the transferability of previously reported T2D SNPs in the AADM sample. Of the 130 SNPs, 108 were present in our dataset. Sixteen SNPs (or 15%) showed exact replication, i.e., consistent direction of effect for the alleles and *p* < 0.05—Table 3. Two other SNPs [rs2258238 (*HMGA2*) and rs12595616 (*PRC1*)] showed a *p* < 0.05 with inconsistent direction of effect. Sixteen other loci showed local replication, including *KCNJ11*, *HHEX/IDE*, *THADA*, *MC4R*, and *ATP8B2* (Supplementary Table 3). Of the two loci first reported in an African ancestry GWAS meta-analysis for T2D (the MEDIA Consortium), we found exact replication for *INS-IGF2* rs3842770 (*p* = 1.867 × 10^−2^, Table 3), but no evidence for replication for *HLA-B* rs2244020 (rs74995800, *p* = 0.798).

**Table 3:** Exact replication of established T2D loci in 5231 sub-Saharan Africans

SNP	CHR	BP	EA	NEA	EAF	β	SE	*p* Value	Locus
rs7903146	10	114758349	T	C	0.331	0.351	0.049	7.29E−13	*TCF7L2*
rs10998572	10	70859204	A	C	0.055	−0.245	0.105	1.933E−02	*VPS26A*
rs231360	11	2692249	T	C	0.620	0.145	0.048	2.378E−03	*KCNQ1*
rs441613	11	2910191	C	T	0.605	0.115	0.047	1.451E−02	*KCNQ1*
rs2258238	12	66221060	T	A	0.367	−0.101	0.048	3.738E−02	*HMGA2*
rs12595616	15	91563513	C	T	0.852	−0.227	0.066	5.438E−04	*PRC1*
rs1558902	16	53803574	A	T	0.050	0.245	0.105	1.973E−02	*FTO*
rs2925979	16	81534790	C	T	0.676	−0.107	0.049	2.950E−02	*CMIP*
rs7224685	17	4014384	T	G	0.435	0.147	0.046	1.402E−03	*ZZEF1*
rs13342692	17	6946287	C	T	0.393	0.145	0.047	2.009E−03	*SLC16A11/A13*
rs7234111	18	7067652	C	T	0.584	0.0948	0.047	4.433E−02	*LAMA1*
rs4402960	3	185511687	T	G	0.570	0.116	0.047	1.369E−02	*IGF2BP2*
rs1182436	7	157027753	C	T	0.326	0.104	0.050	3.779E−02	*MNX1*
rs3802177	8	118185025	A	G	0.038	−0.311	0.118	8.654E−03	*SLC30A8*
rs10757282	9	22133984	C	T	0.207	0.149	0.056	7.372E−03	*CDKN2A/B*
rs1575972	9	22301092	A	T	0.320	−0.196	0.049	6.47E−05	*DMRTA1*
rs10758593	9	4292083	A	G	0.512	0.117	0.046	1.126E−02	*GLIS3*
rs3842770	11	2178670	A	G	0.291	0.124	0.053	1.867E−02	*INS-IGF2*

*EA* effect allele, *NEA*noneffect allele

### Meta-analysis and transethnic meta-analysis

We conducted an African ancestry T2D meta-analysis that included this GWAS and an African American meta GWAS^7^ consisting of five studies (*n* = 8599); the Atherosclerosis Risk in Communities (ARIC), the Cleveland Family Study (CFS), the Howard University Family Study (HUFS), Jackson Heart Study (JHS), and Multi-Ethnic Study of Atherosclerosis (MESA). This African ancestry meta-analysis revealed four genome-wide significant loci (Supplementary Fig. 4, Supplementary Table 4), namely *TCF7L2* (lead SNP rs386418874 *p* = 6.33 × 10^−11^), *TH-INS* (lead SNP rs4072825 *p* = 2.35 × 10^−8^), *KCNQ3* (lead SNP rs111248619 *p* = 2.71 × 10^−8^), and an intergenic locus (lead SNP rs4532315 *p* = 9.80 × 10^−9^). *TCF7L2* and *TH-INS* are well-known T2D loci and *TH-INS* has also been reported to be associated with other forms of diabetes (maturity-onset-diabetes of the young and transient neonatal diabetes) and other metabolic phenotypes. *KCNQ3* (potassium voltage-gated channel subfamily Q member 3) encodes a potassium voltage-gated channel which regulates neuronal excitability. Defects in the gene are a cause of a form of neonatal epilepsy (benign familial neonatal convulsions type 2 or BFNC2), but variants in the gene have not been associated with T2D or other metabolic phenotypes. The chromosome 5 intergenic locus with lead SNP rs4532315 has not been shown to be associated with any phenotype so far.

Transethnic meta-analysis of the above African ancestry studies with a large GWAS of European ancestry individuals (the DIAGRAM meta-analysis of type 2 diabetes (T2D) based on the GoT2D integrated haplotypes)^8^ revealed multiple genome-wide significant loci as expected, including *TCF7L2*, *KCNQ1*, *FTO*, *IDE*, *IGF2BP2*, *CDKAL1*, and *SLC30A8*. However, they are all known loci, and none is a novel locus for T2D.

### Suppression of zebrafish zranb3 results in reduced β-cell mass

To examine a potential role for *zranb3* in T2D etiology in vivo, we carried out functional studies of the zebrafish ortholog, *zranb3*, at larval stages. We first examined embryonic expression of the gene by RNA-seq in pancreatic β-cells isolated by FACS from 5 dpf (days postfertilization) wild-type larvae, a stage at which the larval β-cells are responsive to glucose and other nutrients and also exhibit calcium oscillations indicative of a functioning islet^9,10^. In comparison to other known β-cell genes which were highly expressed, we also detected expression of *zranb3* in β-cells by RNA-seq, a finding which we did not observe for markers of other tissues including heart and bone (Supplementary Fig. 5a). This finding was confirmed by quantitative polymerase chain reaction (qPCR) (Supplementary Fig. 5b).

**Fig. 3:**
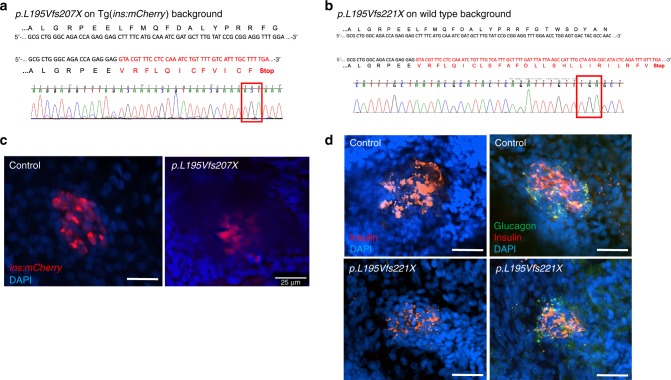
Generation of *zranb3* genomic zebrafish mutants. **a**, **b** Identified mutations found in homozygous F2 animals by CRISPR/Cas9 in Tg(*ins:*mCherry*)* or wild-type animals. In both lines genomic DNA deletions in exon 4 resulted in frame shift and premature stop codons (red box), confirmed by sequencing. **c** Confocal microscopy images of *ins:*mCherry transgene (red) in principal islets of wild type or mutant 5 dpf larvae. **d** Confocal images of whole-mount immunostained islets of wild type or mutant 5 dpf larvae, using antibodies against insulin (red) or glucagon (green). Scale bars = 25 µM

Next, we examined a role for *zranb3* in the production or maintenance of β-cells. We targeted the gene in transgenic zebrafish larvae in which β-cells could be visualized and quantified, Tg(*insa:mCherry*)^11^. The zebrafish ortholog was targeted by two approaches. We generated a model of genomic disruption of *zranb3* by CRISPR/Cas9-mediated targeting of exon 4 of *zranb3*. Tg(*insa:mCherry*) embryos co-injected with multiple *zranb3-*targeted short guide RNAs (sgRNA) and Cas9 mRNA were cultured until 5 dpf and β-cell numbers were quantified by identifying locations of intracellular mCherry+. We observed a significant reduction in β-cell numbers in F0 animals injected with sgRNA (28.5 compared with 33.5 in controls, *p* = 0.0006, *t* test). To confirm that these effects were due to heritable genomic disruption of *zranb3*, we propagated progeny of sgRNA-injected animals of either the Tg(*insa:mCherry)* or wild-type background by out-crossing the F0 mutation-carrying fish with wild-type fish to generate F1 heterozygous fish which we then in-crossed to generate F2 homozygous mutants. In both lines, we identified fish homozygous for a deletion and early stop codons (Fig. 3a, b). We examined the F2 mutants in both lines for β-cell mass using either visualization of mCherry in the transgenic line or immunostaining for insulin in the wild-type line. We found that genomic mutation of the *zranb3* gene in zebrafish larvae results in an observable reduction in β-cells, identified as either transgenic *ins:mCherry* expression or the area of insulin+ antibody staining (Fig. 3c, d). In F2 mutants (*n* = 3), we found a mean of 23.0 (SD = 1.63) β-cells compared to wild-type age-matched control larvae (*n* = 15) in which we found a mean of 33.47 (SD = 0.87) β-cells (*t* test with unequal variances *p* = 0.0057). This reflects a mean reduction of 30% in F2-mutant β-cells compared with wild-type age-matched control larvae. Notably, we did not find observable changes in the domain of glucagon expression, detected by immunofluorescent antibody staining (Fig. 3d). No developmental or morphological defects were observed in the mutant larvae at any of the experimental stages.

**Fig. 4:**
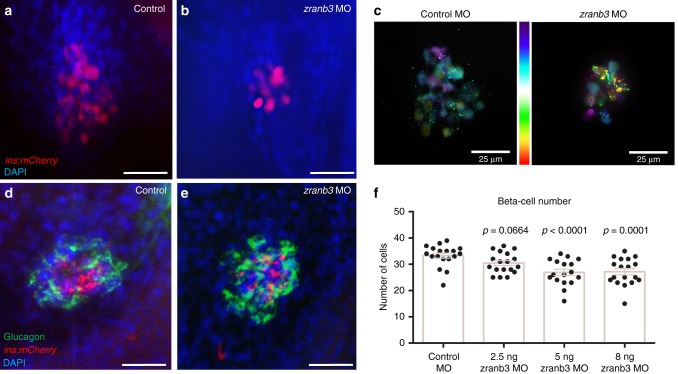
Knockdown of *zranb3* reduces β-cell number in zebrafish larvae. Injection of antisense oligonucleotide morpholinos (MO) targeting *zranb3* reduced β-cells in 5 dpf larval zebrafish, detected by Tg(*ins*:mCherry) expression imaged via whole-mount confocal microscopy (**a**, **b**). **c** Depth coding of confocal microscopy images of whole-mount islets reveals individual cell resolution of β-cells in 5 dpf larval islets, color coded by depth (distal to proximal) along the *z*-axis. **d**, **e** Whole-mount immunostaining of MO-injected Tg(*ins*:mCherry) larval islets using antibody against glucagon (green). **f** Quantification of β-cell number in 5 dpf larval islets of Tg(*ins*:mCherry) injected with indicated concentrations of MO against *zranb*. (*n* = 17–19). Bars represent average across groups. Error bars represent SEM. *p* Values are from the *t* test. Scale bars = 25 µM

To validate these observations, we also injected transgenic one-cell embryos with a morpholino (MO) designed to disrupt splicing of the endogenous *zranb3* transcript at exon 4. We first validated the efficacy of the MO to significantly suppress *zranb3* mRNA expression without inducing off-target toxicity by assessing transcript levels of endogenous *zranb3* and by examining the presence of a marker of MO-induced toxicity, the delta113 isoform of *p53*^12^, respectively (Supplementary Fig. 6A). We cultured injected embryos to 5 dpf and we quantified β-cell number. Quantification by counting of mCherry-expressing cells in larvae with suppression of *zranb3* expression identified a significant reduction of β-cells (26.8 β-cells per larva compared with 33.3 in control larvae, *p* = 0.0001, *t* test; Fig. 4). Quantification of β-cells identified using epifluorescence was confirmed by confocal microscopy and pseudocolor-coding of whole-mount islets to resolve individual cells within the primary islet (Fig. 4c). These findings were consistent with the observations in the CRISPR/Cas9 targeted embryos, suggesting that MO-induced β-cell phenotypes are likely due to disruption of zranb3 expression. The MO-induced effect was directly relevant to knockdown as the impact on β-cell number increased with increased MO dose (Fig. 4f). No developmental morphological defects were observed in the morphants at any dose and at all stages assayed (Supplementary Fig. 6B). Importantly, *zranb3* loss appeared to specifically impact β-cells because we found no significant change in expression of glucagon (Fig. 4d, e). Moreover, we did not detect differences in glucose uptake in peripheral tissues in these animals as determined by treatment with fluorescent 2-NBDG and quantification of retinal fluorescence^13^ (Supplementary Fig. 7).

**Fig. 5:**
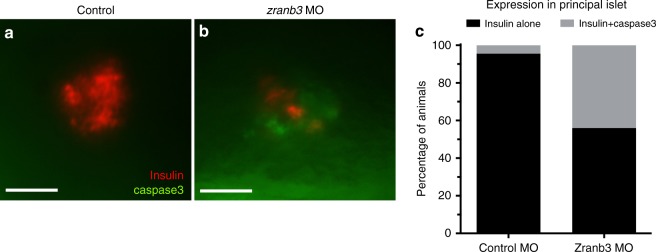
Increased islet apoptosis with loss of *zranb3*. **a**, **b** Co-localization in larval islets of activated Caspase-3 with insulin by whole-mount double immunostaining demonstrating significantly higher proportion of embryos exhibiting co-localization in larval (5 dpf) islets compared to controls, as quantified in (**c**). Data indicate average of three replicate experiments. Each experiment is *n* = 8–10 embryos

To further characterize the role of *zranb3* on β-cell numbers, we examined both proliferation and apoptosis. We assessed proliferation in *zranb3*-deficient Tg(*insa:*mCherry) embryos using FACS analyses and mCherry+ expression to identify β-cells. Cell cycle analysis was completed using DNA content quantification. We found similar proportions of β-cells in G1, S, and G2/M phases as compared to control β-cells, suggesting no significant change in proliferation of these cells (Supplementary Fig. 8). We then asked whether the reduction in β-cell number may be attributable to increased cell death in β-cells. To test this, we used whole-mount immunostaining of zebrafish larvae using an antibody against activated caspase-3, a marker of apoptosis which can be observed in zebrafish larval β-cells^11^. Compared to control larvae in which we found only 4% of larvae expressing caspase-3 in mCherry-positive islets, we found caspase-3 co-localization with mCherry in 44% of *zranb3-*depleted larvae (Fig. 5, *p* < 0.0001, chi-square test), consistent with an increase in islet apoptosis accompanying knockdown of *zranb3*.

**Fig. 6:**
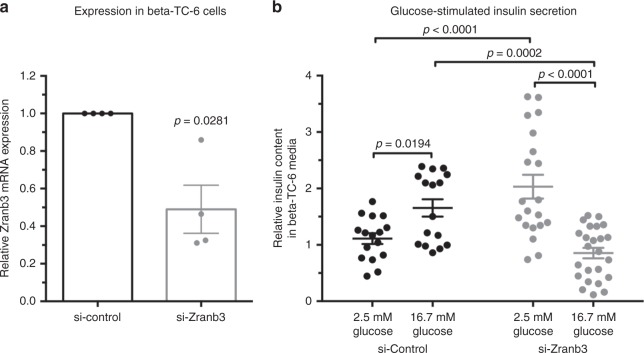
Suppression of Zranb3 in MIN6 β-cells impairs insulin secretion in response to glucose. **a** Reduced *Zranb3* expression upon treatment of MIN6 cells with siRNA targeting *Zranb3*; *n* = 4 independent experimental replicates for each, each dot representing independent experiment and bars represent average values across experiments. **b** Quantification of total insulin secreted by MIN6 cells into culture media upon low- (2.5 mM) and high-(16.7 mM) glucose conditions. *n* = 16–24 replicates for each experiment and averages across experiments indicated. Error bars represent SEM

Our observations of reduced β-cell number due to apoptosis may be consistent with dysfunctional β-cells. To test this possibility, we turned to cultured mammalian β-cells and asked whether suppression of *Zranb3* impacts function. Upon transfection of siRNA specifically targeting murine *Zranb3* (Fig. 6), we treated *MIN6* β-cells with low- or high-glucose conditions and quantified total insulin secreted into the culture media after 1 h. We found that, whereas control cells exhibited a marked increase in insulin secretion upon treatment with high-glucose media, cells transfected with si*Zranb3* did not increase the amount of insulin secreted over the basal amount secreted in low-glucose media (Fig. 6). These observations suggest a necessary role for *Zranb3* in β-cell functional response to high-glucose conditions.

### Integrative analysis of GWAS with transcriptomic data

**Fig. 7:**
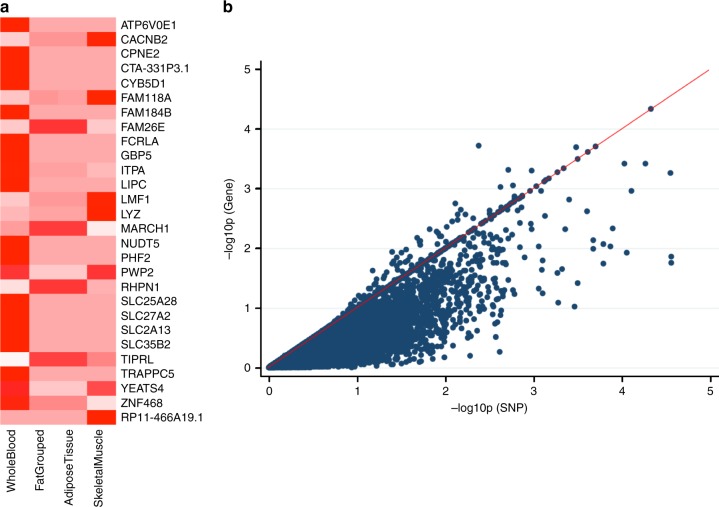
Significant genes on integrative GWAS and transcriptomic analysis. **a** Displays genes with gene-set *p* < 10^−3^ colored in deep red. Transcriptomic data from whole blood (11 studies), “Fat Grouped” (Grundberg et al.^73^ and GTeX adipose tissue), adipose tissue (GTeX adipose tissue only), skeletal muscle (GTeX skeletal muscle only). **b** Plot of gene-based versus best single variant association *p* values for whole blood

Integrative analysis that combines GWAS summary statistics with eQTL data were performed to identify potential new candidate genes. The most significant genes are shown in Fig. 7. Most of these genes have not been reported as T2D-risk loci except for *MARCH1* which associated with T2D^14^. *LIPC* which is associated with closely related phenotypes, including the metabolic syndrome^15^, and circulating levels of total cholesterol, HDL-cholesterol and triglycerides in multiple studies^16–22^.

## Discussion

The vast diversity of genetic characteristics and environments across the world indicates that common complex disorders such as T2D need to be studied in diverse global populations. Nowhere is this truer than in sub-Saharan Africa, which is not only the cradle of humanity but is also home to a vast diversity of populations with widely divergent lifestyle, behavioral and environmental factors including long term exposure to pathogens that have shaped the genomic architecture of African peoples. In the present study, we report a genome-wide analysis of T2D in over 5000 sub-Saharan Africans from a single diabetes association study conducted on the continent. Reassuringly, *TCF7L2* rs7903146 was genome-wide significant as expected from previous T2D studies in Africa^4,23–28^, and consistent with the findings of most populations studied around the world. We also replicated several previously reported T2D loci. Using exact replication strategies (same SNP, consistent direction of effect, *p* value < 0.05), we demonstrated that 15% of the reported loci were significantly associated in this study of sub-Saharan Africans. This is consistent with our estimates of the power of our study to replicate known loci considering effect allele frequencies and reported effect sizes. Using local replication strategies, which help to identify significant loci which would otherwise have been missed because of allele frequency and/or LD differences between populations, we replicated additional sixteen loci (i.e., significant *p* value in a SNP in LD with the original reported SNP). These findings extend our previous studies of transferability of T2D loci in sub-Sharan Africa^4^. Notably, the present study’s exact replication rate of 15% is marginally more than the 11% that we previously reported in a smaller sample from the same study. While this difference is not statistically significant (*p* = 0.369, test of difference between proportions), it suggests that larger sample sizes may lead to increased numbers of replicated variants because of increased statistical power. It should be noted that transferability of reported T2D genome-wide significant variants between populations has always been demonstrable for a relatively small fraction of all such loci, especially with African ancestry populations^13,29^. Perhaps this is not unexpected given that transferability is affected by several factors, including sample sizes, effect allele frequencies, LD structure, and genetic architecture of the trait. While acknowledging that increased sample size is just one of these variables, an appreciation of the final set of consistently replicable variants across populations will probably become possible when sample sizes (and the resultant statistical power) in non-European studies begin to approach those of European GWAS.

A notable finding in the present study is the identification of a previously unidentified locus for T2D, namely *ZRANB3* (Zinc Finger RANBP2-Type Containing 3). Two intronic SNPs in the gene were genome-wide significant and the direction of effect was consistent for the top four SNPs in a South African Zulu sample with the meta-analysis *p* values of the two SNPs remaining genome-wide significant. The SNPs are African-specific and were discovered by sequencing of African genomes in the African Genome Resources Haplotype Reference Panel. Our functional annotation of the *ZRANB3* locus identified several *cis*- and *trans*-eQTLs, indicating that the locus contains multiple functional variants. Of relevance to T2D is our findings of two known-T2D associated genes associated with *cis*-eQTLs (*MCM6*, *DARS*) and four with *trans*-eQTLs (*DGKB*, *GTF3AP5-AGMO*, *IL23R/IL12RB2*, *SLC44A4*). The *DGKB*/*GTF3AP5-AGMO* region has the most annotation to GWAS hits with variants in the genes showing genome-wide significant associations with T2D^30,31^, fasting plasma glucose (FPG) traits^32–34^ and glycated hemoglobin^35^. *IL23R/IL12RB2* is a known GWAS locus for age of onset of T2D^36^, while variants in *SLC44A4* have been implicated in the interaction between T2D and iron status biomarkers^37^. Variants in *MCM6* and *DARS* were recently shown to be associated with total cholesterol change in response to fenofibrate in statin-treated T2D^38^.

Our functional assays in zebrafish focused on the role of the *ZRANB3* ortholog in the pancreas, one of the key tissues in T2D. RNA-seq expression datasets of isolated β-cells showed expression of *zranb3* in the principal zebrafish pancreatic islet. Knockdown of the gene led to reduced *zranb3* expression and to reduction in pancreatic β-cell number in the developing organism, which we confirmed in progeny of animals having nonsense mutations introduced into *zranb3* by CRISPR. These observations are consistent with recent evidence suggesting that *Zranb3* is highly expressed in replicating murine β-cells^39^, suggesting a likely critical role of the gene in production or maintenance of β-cells. The reduction in zebrafish β-cell mass that we observed was shown to be due to a reproducible increase in apoptosis present in islets of animals with *zranb3* knockdown. The effect of *zranb3* deficiency in the pancreas seems to be specific to β-cells as glucagon in islets is similar between knockout/knockdown and wild-type zebrafish. Glucose tolerance/uptake studied in the animals did not show a defect in glucose uptake that would indicate changes in insulin sensitivity or glucose disposal. We also found that *Zranb3* knockdown in a mammalian cell line (MIN6 β-cells) resulted in an impairment of secretion of insulin in response to high glucose. Overall, the findings from these experiments support an important role for *ZRANB3* in T2D that is mediated through a mechanism of impaired insulin response at the level of maintenance and function of β-cell mass. These findings in combination with the eQTL findings suggest that the *ZRANB3* locus may act directly, through other loci that it regulates (e.g., *DGKB*, *GTF3AP5-AGMO*, and *IL23R/IL12RB2*) or in combination with those loci to produce the pathophysiological changes that lead to altered glucose metabolism and T2D.

Integrative analysis of GWAS and transcriptomic studies are increasingly being utilized to identify novel candidate genes which may not have been detected through either type of study alone as illustrated by loci above the null line and towards the upper right quadrant of Fig. 7b. We utilized this approach to generate new leads for further studies. We found some candidate genes of which two (*MARCH1* and *LIPC*) are established T2D-related loci. The fact that they were not significant in our study indicates that integrative analysis can boost the capacity of a GWAS to identify and/or prioritize loci for further study. One of the major drawbacks of this type of integrative analysis is the relatively small sample sizes of most transcriptomic datasets^40^, which limits the power of the eQTL studies. However, this limitation would gradually diminish as more data are generated.

The identification of a previously unidentified candidate T2D locus in the present study provides further support for the notion that genome analysis studies in diverse global populations have the potential to discover novel risk loci and improve our knowledge of the genetic architecture of many common complex disorders^41–45^. For T2D, this has been demonstrated in studies which identified *SLC6A11* in Mexicans by the SIGMA Type 2 Diabetes Consortium^46^, *SGCG* in Punjabi Sikhs^47^, and *KCNQ1* in East Asians^48,49^ as novel risk loci for T2D. In the search for novel loci, this strategy of including populations of different ancestries complements the strategy of increasing sample sizes to boost statistical power to detect small effect sizes.

The present study addressed discovery science in the context of under-represented populations in genomic research, partly in response to the lack of diversity and predominance of European ancestry populations in genomic studies^42,44,50–52^. Several examples now exist for how lack of diversity in genomic studies is resulting in missed opportunities for discoveries and for more robust understanding of heterogeneity in effect sizes across ethnic groups. A recent example from the Population Architecture using Genomics and Epidemiology study demonstrated that one-quarter of genetic associations in the NHGR-EBI GWAS Catalog show significant heterogeneity in effects sizes between ethnicities^53^. Given that effect sizes are estimates of risk, this implies that risk prediction would vary substantially depending on the ethnic group. It is important to recognize that the effects of this lack of diversity extend beyond discovery science to translational studies because the resulting gaps in knowledge may lead to missed opportunities for developing clinical guidelines, better tailoring of clinical guidelines and treatment protocols and developing new therapeutic agents^45,54^. Highlighting these issues further is the recent call for better calibration of polygenetic risk scores (PRS) to enhance transethnic utility with the hope of not exacerbating already unacceptable health disparities as PRS is used to identify high-risk individuals for early intervention in clinical and public health settings^55,56^.

The strengths of the present study include a relatively large sample size, a focus on an understudied ancestral group, use of state-of-the-art SNP microarrays and imputation to an African enriched reference panel providing an unparalleled comprehensive opportunity to test millions of common SNPs across African genomes. A potential limitation is that SNPs with small effect sizes are not detectable with the present sample size. More studies in Africans and combined analysis using meta-analytic procedures would overcome this limitation.

In summary, this GWAS of T2D in over 5000 sub-Saharan Africans replicated several known T2D loci, including *TCF7L2* and identified *ZRANB3* as a T2D locus. Functional experiments in zebrafish suggest that *ZRANB3* is important in β-cell mass, and thereby the capacity of the pancreas to respond to insulinogenic stimuli.

## Methods

### Ethics statement

All human research was conducted according to the Declaration of Helsinki and all relevant ethical regulations for work with human participants. The study protocol was approved by the institutional ethics review board (IRB) of the National Institutes of Health/National Human Genome Research Institute (protocol HG-09-N070) and the IRBs of each participating institution, including University of Lagos, Lagos, Nigeria; College of Medicine, University of Ibadan, Ibadan, Nigeria; University of Nigeria Teaching Hospital, Enugu, Nigeria; University of Science and Technology, Kumasi, Ghana; University of Ghana Medical School, Accra, Ghana. Written informed consent was obtained from each participant prior to enrollment.

All experiments performed on animals have been approved by the University of Maryland Baltimore IACUC under protocol approval number 1218020.

### Study participants

Study participants are from the Africa America Diabetes mellitus (AADM) study^3,6^. This is a study of the genetic epidemiology of T2D in Africa that enrolled participants from Nigeria, Ghana, and Kenya. The study eligibility criteria and enrollment procedures have been described in detail elsewhere^3,4,6^. Briefly, participants were Africans enrolled through major medical centers in Nigeria (Ibadan, Lagos, and Enugu), Ghana (Accra and Kumasi), and Kenya (Eldoret). The most common ethnolinguistic groups in the study sample were Yoruba and Igbo (Nigeria); Akan and Gaa-Adangbe (Ghana); and Luhya, Kikuyu, and Kalenjin (Kenya). Participants identified at each center were first consented for the study and underwent the same enrollment procedures, which included collection of demographic details, medical history, and clinical examination. Clinic procedures included anthropometry for weight, height, waist circumference, and hip circumference; three blood pressure measurements in the sitting position; and collection of fasting blood samples. Weight and height were measured in light clothes. Weight was measured to the nearest 0.1 kg using an electronic scale while height was measured to the nearest 0.1 cm with a clinical stadiometer. BMI was calculated using the formula: weight (kg)/height^2^ (m).

The case definition of T2D was done using the criteria of the American Diabetes Association (ADA). The criteria were a FPG concentration ≥ 7.0 mmol/L (126 mg/dl) or an oral glucose tolerance test (OGTT) 2 h postglucose load ≥ 11.1 mmol/L (200 mg/dl) on more than one occasion. A diagnosis of T2D was also accepted if a patient was on physician prescribed pharmacological treatment for T2D and a review of clinical records showed that pre-treatment fasting glucose and/or OGTT criteria was consistent with the diagnosis. To exclude probable cases of type 1 diabetes, patients with autoantibodies to glutamic acid decarboxylase and/or a fasting C-peptide ≤ 0.03 nmol/L were excluded. T2D controls were required to meet the following criteria: FPG < 6.1 mmol/L (110 mg/dl) or OGTT 2 h postglucose load < 7.8 mmol/L (140 mg/dl) and must have none of the classical symptoms of diabetes (polyuria, polydipsia, and unexplained weight loss).

### Genotyping and imputation

The 5231 samples were genotyped on two platforms: 1808 samples were genotyped using both the Affymetrix Axiom^®^ PANAFR SNP array (an array with ~2.1 million SNPs that is optimized for African ancestry populations (see Web Resources)) and the Affymetrix 319(R) Exome Array and 3423 samples were genotyped using the Illumina Consortium array: Multi-Ethnic Global Array (MEGA) (see Web Resources). Each of the resulting datasets underwent separate quality control. After technical quality control, sample-level genotype call rate was at least 0.95 for all subjects. Each SNP dataset was filtered for missingness, Hardy–Weinberg equilibrium (HWE) and allele frequency. SNP passing the following filters were retained: missingness < 0.05, HWE *p* < 1 × 10^−6^ and minor allele frequency > 0.01. SNPs that passed quality control were used as the basis for imputation. Imputation of all samples was performed using the African Genome Resources Haplotype Reference Panel (a new African genome reference panel based on 4956 samples from all African and non-African 1000 Genomes Phase 3 populations and additional African genomes from Uganda, Ethiopia, Egypt, Namibia, and South Africa) using the Sanger Imputation Service (see Web Resources). The additional African genomes included 2298 African samples with whole-genome sequence data from the African Genome Variation Project (AGVP)^57^ and the Uganda 2000 Genomes Project (UG2G). This new panel both increased the number of imputed variants and improved the information score and imputation accuracy for African populations when compared with the 1000 Genomes Phase 3 Version 5 reference panel. The resulting imputation dataset of all samples was filtered for variants with MAF ≥ 0.01 and information score (info) ≥ 0.3 for association analysis.

### Association analysis

Association analysis was done using the generalized linear mixed model association test (GMMAT) R package^58^, a software package for association tests based on generalized linear mixed models. We computed PCs using an LD-pruned subset of SNPs (Supplementary Fig. 1). Similar to our previous study^4^, we found that the first three PCs were significant and were therefore included in downstream analyses. To account for relatedness between individuals in the sample, we computed a genetic relatedness matrix (GRM) using the Genome-wide Efficient Mixed Model Association algorithm^59^. Association testing for T2D was done using the mixed logistic model as implemented in GMMAT. This is a score test which was done with the imputed genotype dosages with age, gender, BMI, the GRM and the first three PCs as covariates.

### Statistical power estimates

The power of the study for discovery was estimated using *Quanto*^60^ and assuming an *α* of 5 × 10^−8^. For a variant with a minor allele frequency (MAF) of 0.05, the study has 80% power to detect a genetic risk ratio (GRR) of 1.7 and 94% power to detect a GRR of 1.8. For a variant with MAF of 0.10, the study has 82% power to detect a GRR of 1.5 and 98% power to detect a GRR of 1.6.

### Replication of *ZRANB3* in an African ancestry study

To evaluate replication of ZRANB3 in another African population, we examined the significant variants in South African Zulu T2D cases and controls from the Durban Diabetes Study (DDS) and the Durban Diabetes Case–Control Study (DCC). The DDS is a population-based cross-sectional study of nonpregnant urban black African individuals aged 18 years and above living in the city of Durban, South Africa^61,62^. They all belonged to the Zulu ethnolinguistic group and a diagnosis of T2D was based on WHO criteria. Participants in the DCC consisted of South African Zulu patients who were diagnosed with type 2 diabetes based on WHO criteria and were attending a diabetes clinic at either Inkosi Albert Luthuli Central Hospital in Durban, South Africa, or one of three peripheral clinics. A total of 2578 Zulu participants (1602 T2D cases and 976 controls) were included in the replication study.

### eQTL annotation of ZRANB3

For eQTL annotation of the genome-wide significant *ZRANB3* SNPs, we utilized data from the FHS eQTL Study^40^ accessed via the NCBI Molecular QTL Browser (see Web Resources). This is a microarray-based genome-wide study that analyzed both *cis-* and *trans*-eQTLs in whole blood samples from over 5000 study participants. We chose this database because till date it is currently the largest, single site study of both *cis*-eQTLs and *trans*-eQTLs. First, we used the haplotype block definition method of Gabriel et al.^63^ to construct haplotypes around the two genome-wide significant SNPs, resulting in an 18.7 kb haplotype block around 2:136064024 and a 16.8 kb haplotype bock around 2:136019729. Next, we retrieved significant eQTLs in these two haplotype blocks from the FHS-eQTL Study and identified *cis*- as well as *trans*-eQTL SNP-gene pairs. We then overlaid the gene lists from the retrieved eQTL data on the list of significant associations with T2D in the NHGRI-EBI GWAS Catalog. To provide finer resolution, we annotated the SNPs flanking each genome-wide significant *ZRANB3* SNP for eQTLs.

### Meta-analysis

Given the paucity of genome-wide data on Africans characterized for T2D, we conduct a meta-analysis of our GWAS with an African ancestry dataset: a GWAS of African American samples for T2D (*n* = 8599) conducted on African American participants from five studies (ARIC, the CFS, the HUFS, JHS, and MESA) retrieved under controlled access from dbGAP. We used a fixed effects model with inverse weighting of effect sizes as implemented in *METAL*^64^ with double genome inflation correction. As a check, we utilized a meta-analysis method that allows for heterogeneity of effects as implemented in MetaSoft^65^ and obtained essentially the same findings (thus, results from *METAL* are presented). For transethnic meta-analysis, we conduct a meta-analysis for T2D with data from the present GWAS, the African American studies and the DIAGRAM meta-analysis of 13 cohorts imputed from the GoT2D integrated haplotype reference panel^8^.

### Transferability of established type 2 diabetes loci

We looked for evidence of transferability of established T2D loci reported in the literature and curated with the aid of the NHGRI-EBI GWAS Catalog and updated with the latest meta-analysis studies. We considered a *p* value < 0.05 associated with a SNP with the same direction of effect as evidence of transferability. Where the exact SNP was not present or did not show significant association in our dataset, we examined all SNPs with LD *r*^2^ > 0.3 and within +250 kb of the reported index SNP for association with T2D. Nominal association *p* values were adjusted for the total number of SNPs within the region using the method of effective degrees of freedom^66,67^. A locus was considered to show local replication if it had at least one of the tested SNPs with adjusted association *p* value < 0.05.

### Zebrafish lines

Experiments were carried out using Tg(*insa*:mCherry)^68^ or wild-type animals of the Tubingen strain. Adult zebrafish were housed and naturally mated according to standard protocol. All zebrafish work was conducted in accordance with University of Maryland IACUC guidelines.

### MO and CRISPR/Cas9

MO antisense oligonucleotides (MOs) that block splicing (SB) at the splice junction of exon 4 of *zranb3* mRNA were injected into one- to two-cell stage embryos. We designed SB MO (5′-GATACTCCTGCAAAGCAAACAAACA-3′). A control nonspecific MO was used (5′-CCTCTTACCTCAGTTACAATTTATA-3′). The embryos were grown at 28 °C until harvesting for analyses. MO efficacy and off-target toxicity was assessed in cDNA generated from total RNA isolated from homogenates of whole 5 days postfertilization (dpf) larvae and qPCR analysis^11^.

Target sites for CRISPR were determined and designed according to published protocols^11^. We identified target sites within either exon 4 or exon 7 of *zranb3* to which we generated sgRNAs by in vitro transcription using the following oligo sequences:

Exon 4 oligo1: TAATACGACTCACTATAGGATGGCACGCTTGGCGCTCGTTTTAGAGCTAGAAATAGC

Exon 7 oligo 1: TAATACGACTCACTATAGGGAATTCGCTGGCGTATTTGTTTTAGAGCTAGAAATAGC

Universal Oligo 2: AAAAGCACCGACTCGGTGCCACTTTTTCAAGTTGATAACGGACTAGCCTTATTTTAACTTGCTATTTCTAGCTCTAAAAC

The sgRNAs, at 25 pg/µl, along with Cas9 mRNA, at 300 pg/µl, were microinjected directly into the cell during the single cell stage of embryonic development.

### Quantitative RT-PCR

Zebrafish were anesthetized with MS-222, before removing a small section of caudal fin, or grinding up a 24 hpf embryo. RNA was extracted from the sample using Trizol reagent (Life Technologies) according to manufacturer’s protocol and purified using the RNeasy Kit (QIAGEN). cDNA was transcribed using the Fermentas First Strand cDNA Transcription Kit (Thermo Scientific) according to manufacturer’s protocol, diluted to 1∶9 and added to a reaction including target-specific primers (sequences provided in Supplementary Note 1) and LightCycler 480 SybrGreen (Roche) and run on a LightCycler 480 (Roche) for 5 min at 95 °C then 40 cycles of 95 °C (10 s), 58 °C (10 s), 72 °C (10 s) then 5 min at 72 °C. A reverse-transcriptase-free sample was used as a negative control. All samples were run in duplicate with the *C*_T_ value normalized to Actin, RPIII, and/or EF1α to calculate relative expression for each gene in each sample. Biological replicates were repeated.

### Whole-mount immunofluorescence, in situ hybridization, and imaging of larval zebrafish islets

Zebrafish embryos were fixed overnight in 4% paraformaldehyde, washed three times in PBS + Tween20 (0.1%). Embryos were transferred to 150 mM Tris pH9 for 5 min at room temperature, then 15 min at 70 °C. Embryos were then cryoprotected in 30% sucrose overnight at 4 °C and then processed for immunofluorescence or imaged using a Nikon W1 confocal microscope at 60×. Images were compiled and analyzed using Fiji^69^. Immunofluorescent staining was carried as per previous protocols^11^ using activated caspase-3 and insulin antibodies or antibody against glucagon (Sigma, 2654) used at 1:100 concentration.

### Zebrafish β-cell analysis

The Tg (*insa*:mCherry) line which labels β-cells specifically by expressing mCherry under the control of the preproinsulin (*insa*) promoter was used to quantify the number of β-cells according to previous published protocol^11^. Briefly, embryos were fixed in 4% PFA, washed in phosphate buffered saline with Tween-20 and flat mounted in ProLong^**®**^Gold antifade (Life Technologies) with the right lateral side facing the coverslip. Sufficient pressure was applied to disrupt the islets in order to visualize individual cells. The number of β-cells was counted manually under an Olympus IX71 fluorescence microscope and imaged and analyzed using CellSens software. Quantification was confirmed in separate experiments using whole-mount 5 dpf larvae immunostained for insulin and imaged on the Nikon W1 confocal microscope at 60×. Images were compiled and analyzed for depth resolution using Fiji^69^. The analysis of β-cells was performed on embryos collected from three different injections of either control or test morpholinos or embryos generated from mutant adults.

### 2-NBDG uptake

Embryos at 5 days post-fertilization (dpf) were incubated with 0, 300, 600, or 1000 μM 2-(N-(7-Nitrobenz-2oxa-1,3-diazol-4-yl)Amino)-2-Deoxyglucose (2-NBDG, abcam), a fluorescent glucose mimetic, for 6 h at 28.5 °C. Embryos were immobilized using 3-amino benzoic acid ethyl ester (Tricaine) and imaged on a Lionheart FX at 4× (BioTek Instruments). Total retinal fluorescence images were compiled and quantified using Fiji^69^.

### Isolation of zebrafish β-cells and cell cycle analysis

Single-cell dissociation was carried out on Tg(*insa:mCherry*) embryos as per published protocols^70^, and stained for DNA content using DyeCycle Violet (Life Technologies). DNA content analysis was carried out by FACS on a BD LSR II and analyzed using the *FlowJo* software package (FlowJo, LLC).

### FACS-assisted Isolation and Analysis of β-cells

Five days post-fertilization larvae were dissociated into single cells using published methodology^70^. For RNA-Seq analysis, the single-cell suspension was sorted via mCherry+ signal using a BD FACS Aria II (BD BioSciences) and RNA was extracted from isolated β-cell fraction via extraction kit (Qiagen). RNA quantity and quality were assessed via 260/280 absorption. Samples were provided in duplicate for library preparation and quantitative analysis using Next Generation Sequencing and an Illumina HiSeq 2 × 150 PE (GENEWIZ). Fragments were aligned to the GRZ10 genome with CLC Genomics Server program v10.0.1. We used three hundred 5 dpf larvae for sorting and isolated approximately 0.08% of all cells, based on expression of the mCherry reporter gene (i.e., β-cells). In total, 1500–2000 cells were used for generation of the RNA-Seq dataset.

### Cultured β-cells and glucose stimulated insulin secretion

Culture of *MIN6* cells (CRL11506; American Type Culture Collection) were cultured in DMEM-H (American Type Culture Collection) supplemented with 15% heat-inactivated fetal bovine serum and 1× penicillin/streptomycin (Sigma). Knockdowns were accomplished using Lipofectamine 3000 (Life Technologies) and either scrambled control or *Zranb3*-targeted siRNA (Life Technologies). Efficacy of siRNA knockdown was evaluated via qRT-PCR. Glucose stimulation of cultured β-cells was performed on cells plated at equal densities, as determined by hemocytometer counts, using 2.5 and 16.7 mM glucose as baseline and high-glucose concentrations, respectively. Insulin was assessed in media collected at the indicated time points using high-sensitivity insulin ELISA (Mercodia Mouse Insulin ELISA Catalog number 10-1247-10).

### Integrative analysis of GWAS with transcriptomic data

To identify potentially novel candidate genes and generate new hypotheses, we leveraged our GWAS to conduct functional gene set-based analysis of the AADM GWAS summary statistics using publicly available transcriptomic data on selected T2D-related tissues. We conducted gene-based association analysis of eQTLs with the intention of combining genome-wide association statistics with transcriptomic data to co-localize T2D loci which may not have been discovered by using either approach alone. We used a gene-based method instead of a single-SNP method because under the assumption that the expression of a gene is causally-related to disease status and gene expression is determined by multiple independent SNPs, a gene-based test that captures the aggregate effects of these SNPs should have better power over testing each SNP individually. We used *EUGENE* v1.3b^71^ using the AADM GWAS summary statistics as input and accounted for LD between variants with the 1000 Genomes AFR data. We restricted our analyses to adipose tissue, skeletal muscle and whole blood (T2D-related target tissues for which reference eQTL data are available) using precomputed eQTL reference data provided by the software authors. We used Satterthwaite’s approximation to estimate the significance of the gene-based sum statistic (i.e., *GCTA-fastBAT*)^72^, a method which is more efficient than using simulations to estimate the gene-based sum statistic and is recommended as the default approach to estimate significance.

### Web resources

For Affymetrix Axiom^®^ PANAFR array, see https://www.thermofisher.com/order/catalog/product/901788?ICID=search-product; for ClinVar Accession SCV000191187.1, see https://www.ncbi.nlm.nih.gov/clinvar/variation/156804/; for dbGaP, see https://www.ncbi.nlm.nih.gov/gap; for Eugene, see https://genepi.qimr.edu.au/staff/manuelF/eugene/download.html; for GMMAT, see https://content.sph.harvard.edu/xlin/software.html#gmmat; for Illumina Multi-Ethnic Global Array (MEGA), see https://www.illumina.com/science/consortia/human-consortia/multi-ethnic-genotyping-consortium.html; for MetaSoft, see http://genetics.cs.ucla.edu/meta/index.html; for The NHGRI-EBI Catalog of published genome-wide association studies, see https://www.ebi.ac.uk/gwas/; for NCBI Molecular QTL Browser, see https://preview.ncbi.nlm.nih.gov/gap/eqtl/studies/; for OMIM, see http://www.omim.org/; for Sanger Imputation Service, see https://imputation.sanger.ac.uk/.

### Reporting summary

Further information on research design is available in the Nature Research Reporting Summary linked to this article.

## Supplementary information

Supplementary Information

Reporting Summary

## Data Availability

The GWAS summary statistics are available at dbGap [Accession number phs001844.v1.p1] for disease-related research consistent with the ethical approvals governing the study. The RNA-Seq datasets are accessible online at the Gene Expression Omnibus (GEO) under the accession “GSE125354” [https://www.ncbi.nlm.nih.gov/geo/query/acc.cgi?acc=GSE125354]. All other data are contained within the article and its Supplementary Information or upon reasonable request from the corresponding author.
