# Supplementary material for: ZRANB3 is an African-specific type 2 diabetes locus associated with beta-cell mass and insulin response
Source: Nat Commun. 2019 Jul 19;10:3195. doi: 10.1038/s41467-019-10967-7 (PMC6642147; doi:10.1038/s41467-019-10967-7)
Supplement: Supplementary file 1 — Supplementary Information [file 41467_2019_10967_MOESM1_ESM.pdf]

Supplementary Information

ZRANB3 is an African-specific type 2 diabetes locus associated with beta-cell mass and insulin response

Adeyemo et al.

Supplementary Figures

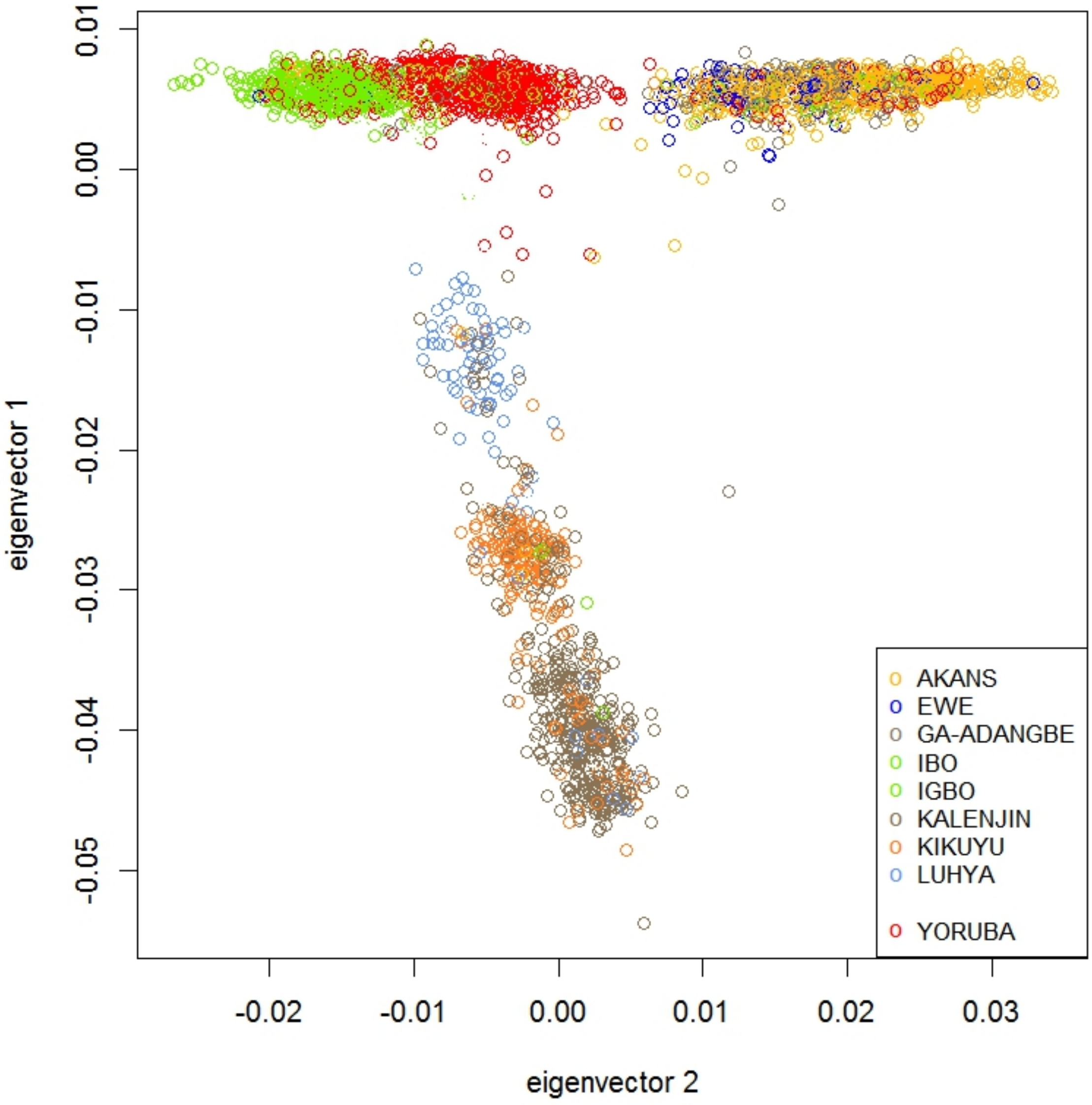

Supplementary Figure 1: Principal Components plot of genotypes of the AADM participants

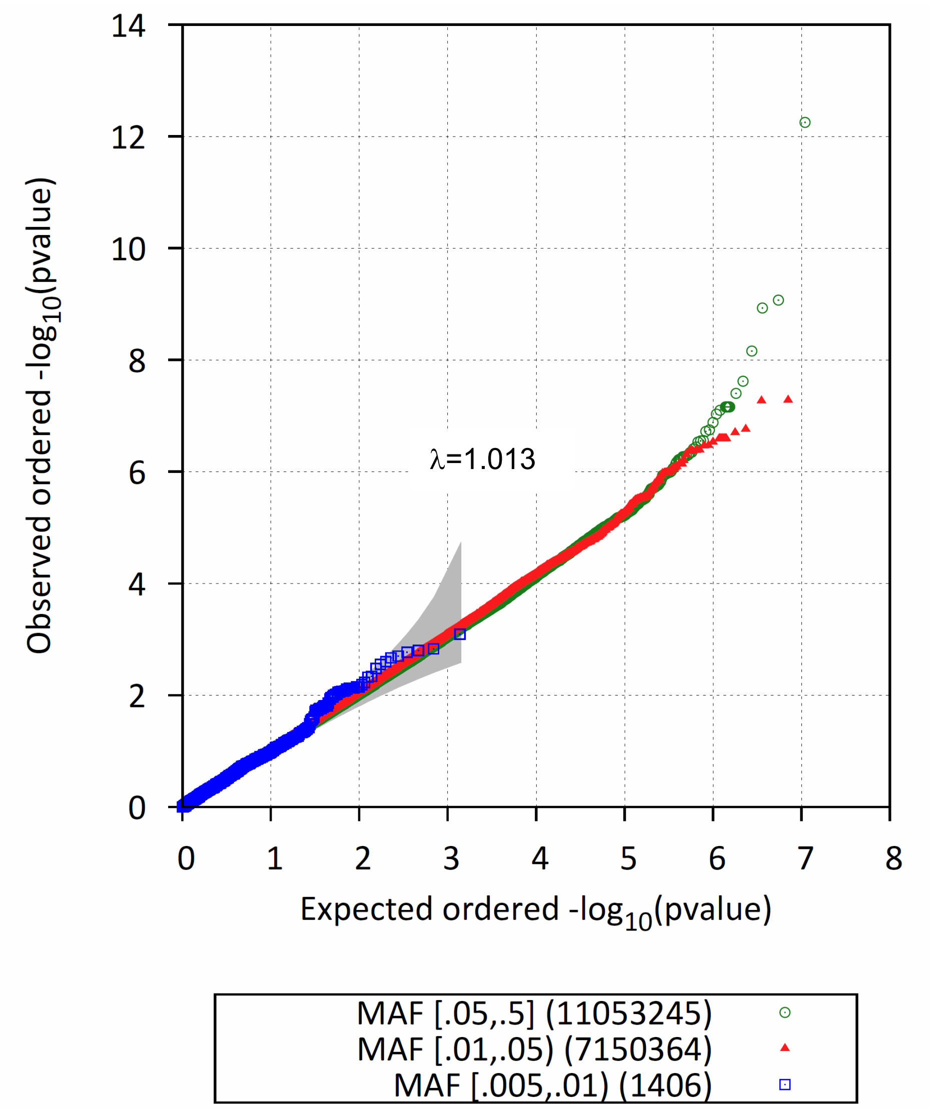

Supplementary Figure 2: QQ plot for the discovery GWAS for T2D: the AADM Study

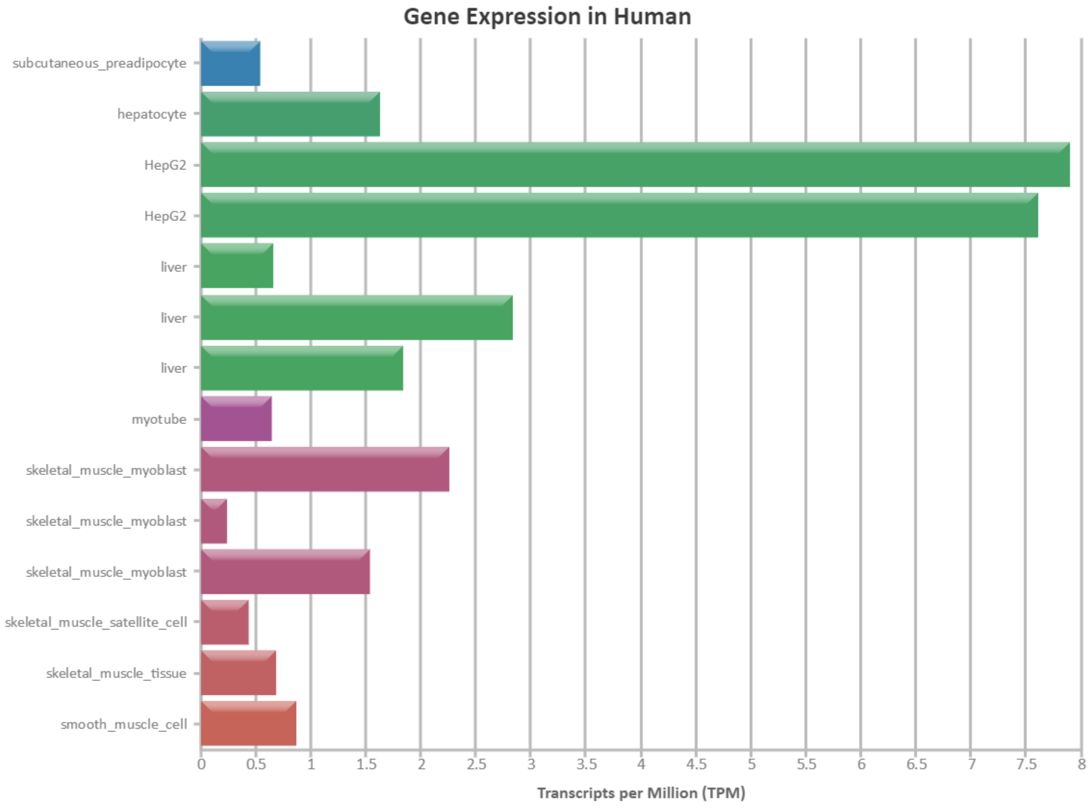

Supplementary Figure 3: ZRANB3 expression in T2D target tissues. Figure generated from ENCODE data

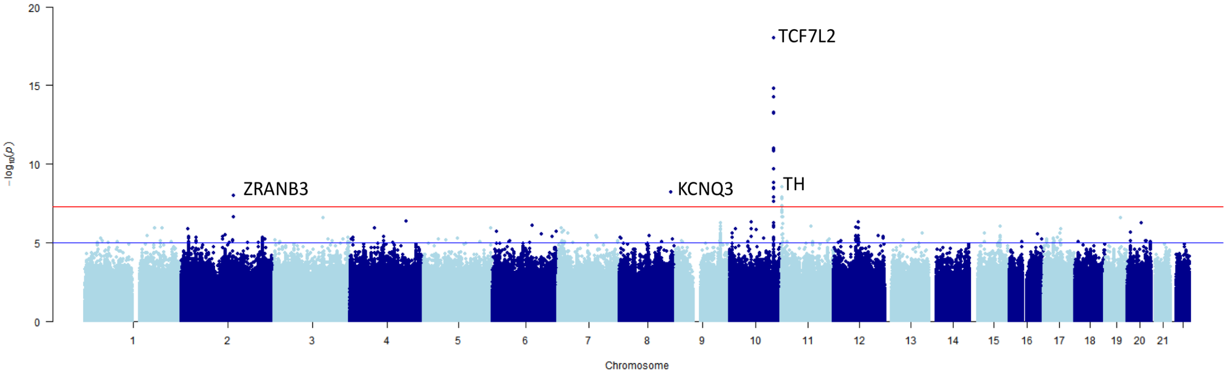

Four genome wide significant loci: *TCF7L2, TH, KCNQ3, ZRANB3*

Supplementary Figure 4: Manhattan plot of meta-analysis of African ancestry - AADM and African American - samples

A

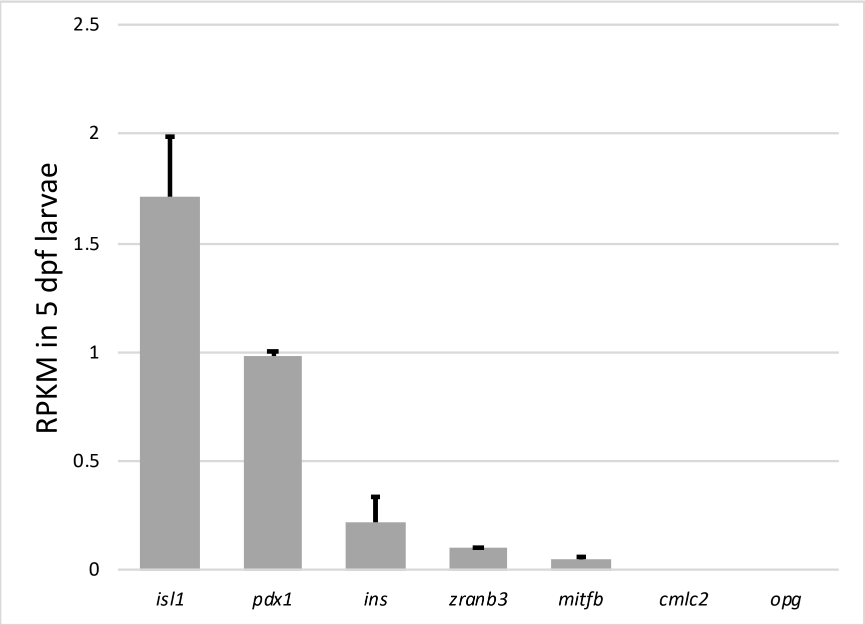

Gene	Mean RPKM	SE RPKM	Annotation of gene
isl1	1.71522249	0.27058771	β-cell gene
pdx1	0.98223831	0.01594826	β-cell gene
ins	0.22035648	0.11522795	β-cell gene
zranb3	**0.1000469**	0.0026255	Gene of interest
mitfb	0.04878491	0.0102144	Skin transcription factor (TF)
cmlc2	0	0	Heart marker by 48 hpf
opg	0	0	Osteoclast differentiation TF

Supplementary Figure 5: Expression of *zranb3* and *selected genes in zebrafish β-cells isolated at 5 days post-fertilization (dpf) A: Expression of zranb3 by RNA-seq in 5dpf zebrafish. Selected genes include both known β-cell genes and genes unrelated to β-cells to give context to *zranb3* expression

B

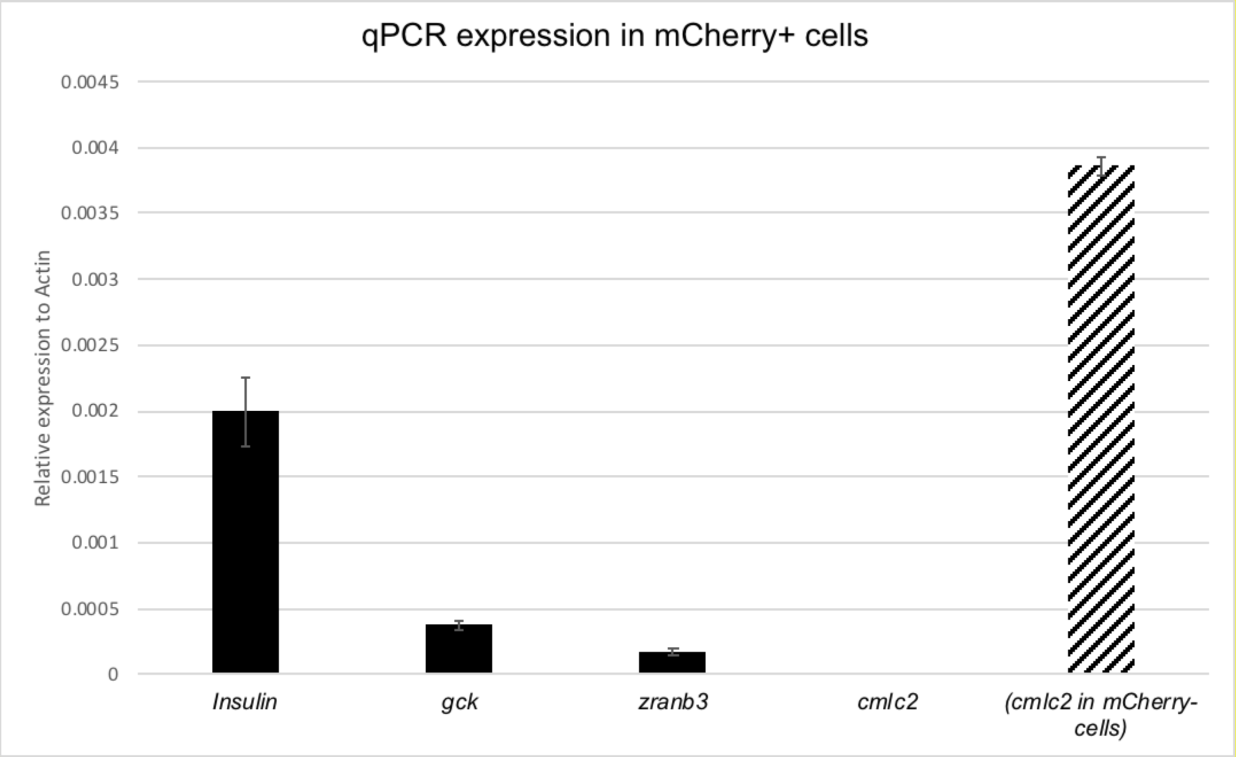

*Relative expression of *cmlc2* in mCherry$^-$ cells shown at far right for reference

**Average values relative to ACTIN**	Relative expression	*Std Dev*
Insulin	0.001995	*0.00026163*
gck	0.000376	*3.53553E-05*
zranb3	0.000167667	*2.08407E-05*
cmlc2	0	*0*
(cmlc2 in mCherry- cells)	0.00386	*7.07107E-05*

Supplementary Figure 5b. *zranb3* expression by qPCR in beta-cells isolated from 5dpf zebrafish*

A

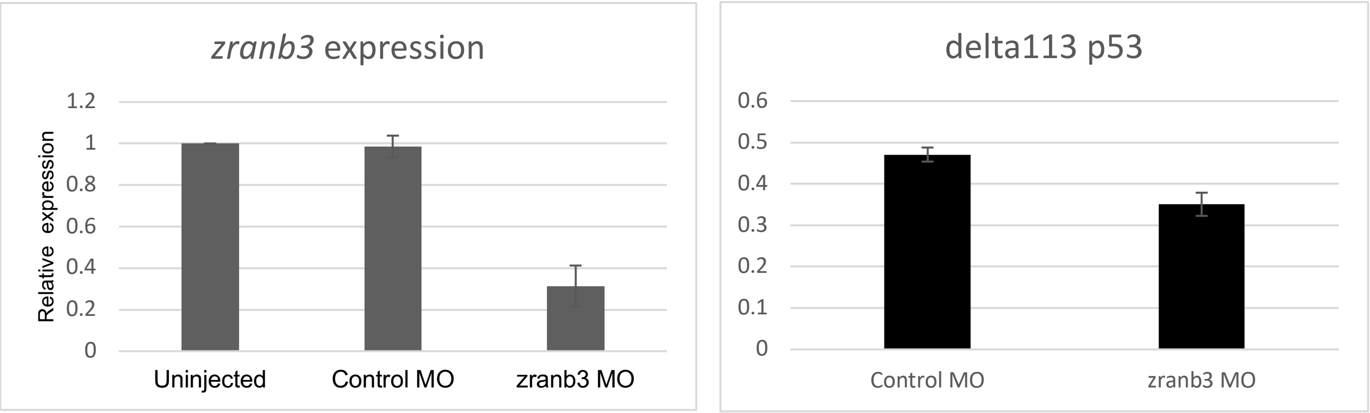

B

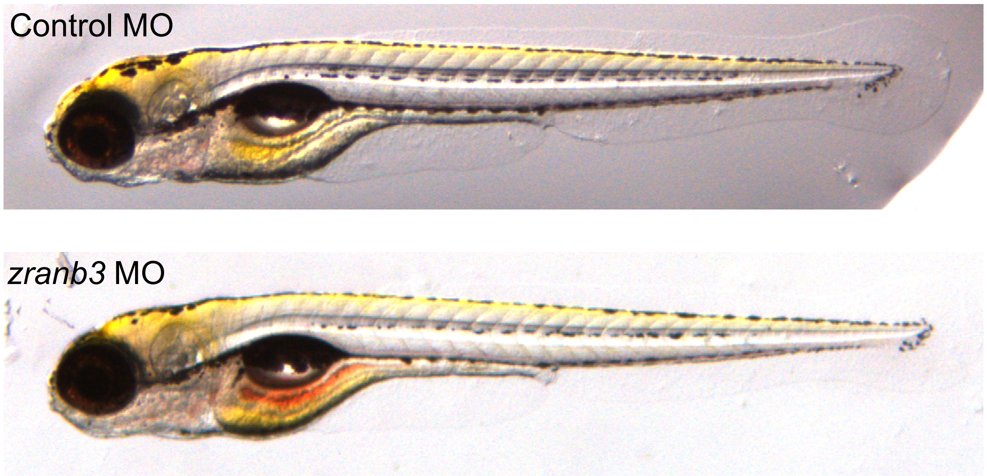

Supplementary Figure 6: (A) Validation of the efficacy of the *zranb3* MO (5ng) to significantly suppress *zranb3* mRNA expression without inducing off-target toxicity evaluated using the presence of a marker of MO-induced toxicity, the delta113 isoform of *p53*. (B) Wholemount bright field images of control MO and zranb3 MO-injected larvae shows no morphological developmental defects at 5 dpf

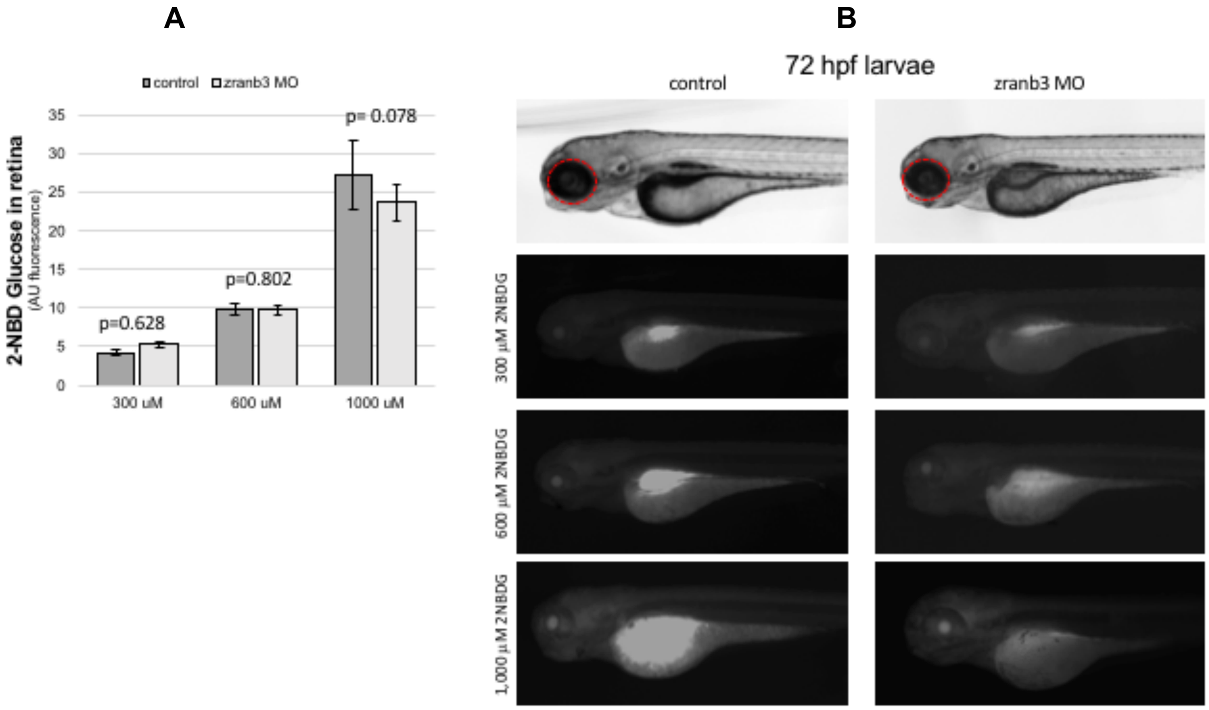

Supplementary Figure 7: Glucose uptake as determined by retinal fluorescence after 2-NBDG treatment. (A) Quantification of glucose uptake at various concentrations in wild-type and *zranb3* MO-injected larvae by quantification of 2-NBDG fluorescence. (B) Fluorescent 2-NBDG staining in control (left panel) and *zranb3* MO-injected (right panel) larvae

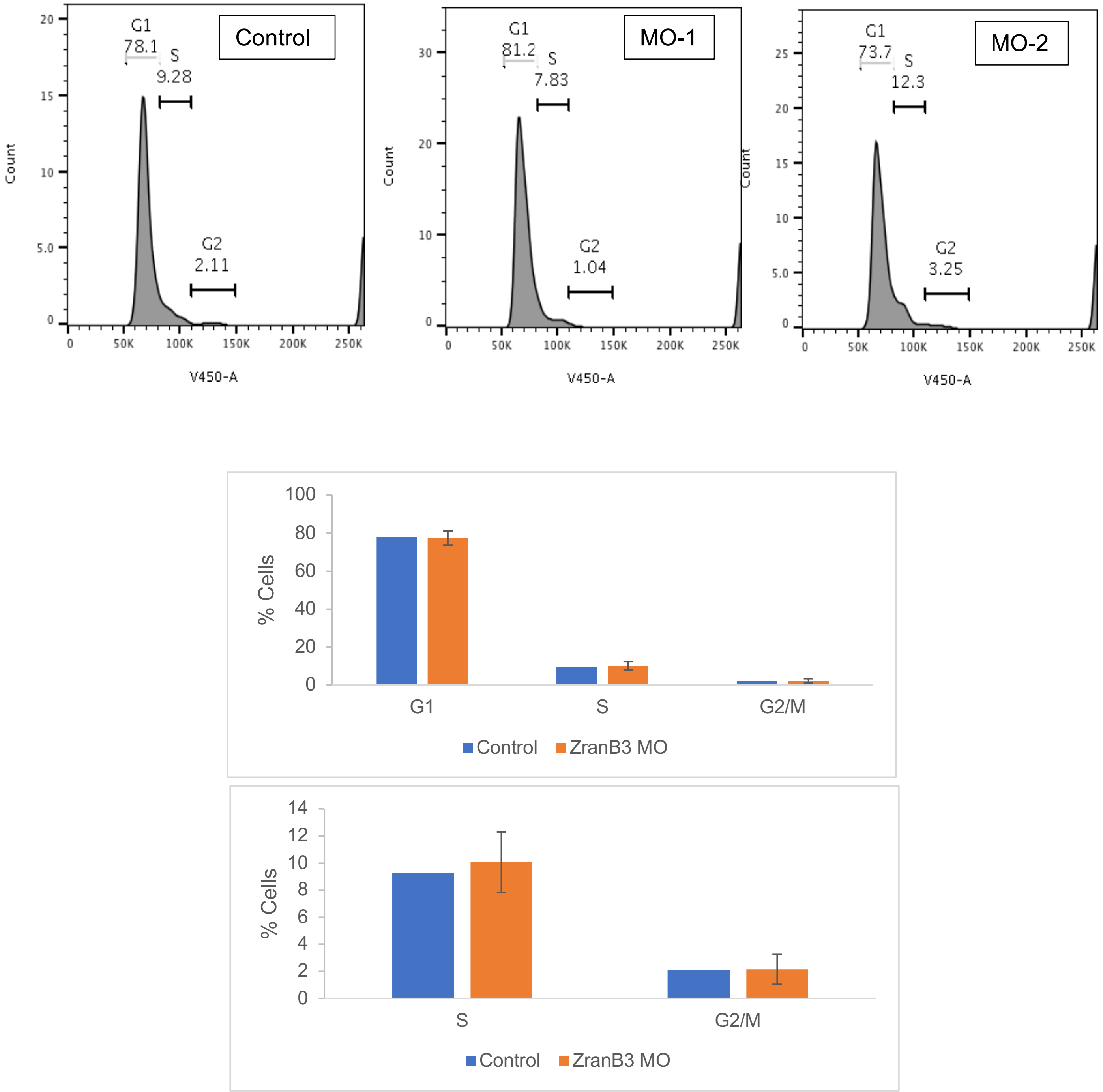

Supplementary Figure 8: Cell cycle analysis of FACS-sorted β-cells control versus *zranb3* MO-injected embryos showing similar DNA histogram profiles (upper panels) for cells from control and *zranb3* MO injected (MO-1, MO-2), quantified in lower panels.

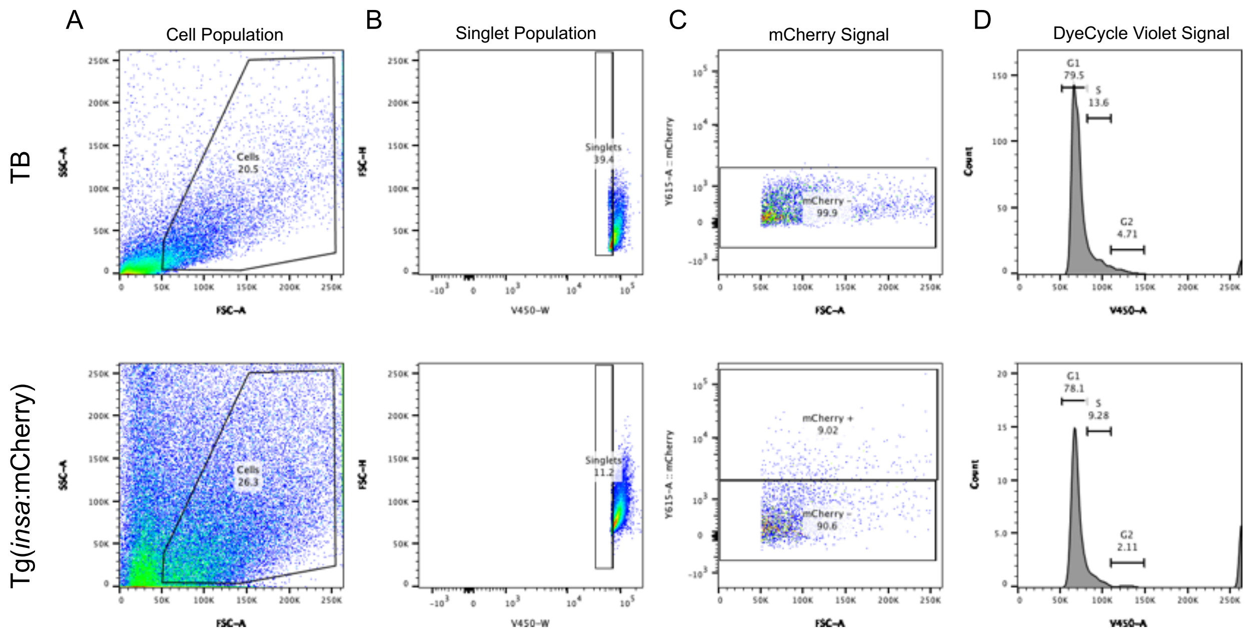

Supplementary Figure 9: Gating strategy for cell cycle determination of zebrafish cells in Tubingen (top) and Tg(*insa*:mCherry) (bottom) animals. **(A)** Gating strategy for cells. **(B)** Gating strategy for single cells. **(C)** Gating strategy for mCherry+ (β) and mCherry- (non-β) cells. **(D)** Gating strategy for G1, S, and G2/M phases of the cell cycle.

Supplementary Tables

Supplementary Table 1: Characteristics of discovery study sample in the AADM Study

Characteristic	T2D	Controls
N	2,342	2,889
Gender (% Female)	58.5	67.4
Age (years)	55.4 (11.0)	45.7 (12.9)
BMI (kg/m2)	26.8 (5.4)	26.5 (6.0)
Waist circumference (cm)	93.7 (11.5)	88.5 (13.2)
Fasting blood glucose (mg/dl)*	153 (109, 232)	85 (79,92)

*Median (interquartile range); all other figures are mean (SD)

Supplementary Table 2: eQTL* annotation of ZRANB3 genome wide significant SNPs in the AADM Study

Index SNP	Haplotype block**			Flanking SNPs			
	***hg19* coordinates (size)**	**Number of target genes for which eQTLs Is in *cis/trans***	**eQTL for T2D-related genes in GWAS Catalog**	**SNP ID**	**Is *cis*-eQTL for *ZRANB3***	**Is *trans*-eQTL (genes)**	**eQTL for T2D-related genes in GWAS Catalog**
2:136064024	2:136046052-136064758 (18.7 kb)	5/43	*MCM6*<br>*DARS*<br>*IL23R/IL12RB2*<br>*SLC44A4*<br>*ZFAT*	rs57510176	Yes	Yes<br>*ATXN10*	-
				rs72982351	Yes	No	-
rs1465146591	2:136016079-136032905 (16.8 kb)	5/35	*MCM6*<br>*DARS*<br>*DGKB*<br>*GTF3AP5-AGMO*<br>*IL23R/IL12RB2*<br>*SLC44A4*	rs141414859	Yes	Yes<br>*DGKB, CGREF1*	*DGKB*
				rs112334908	Yes	No	

*eQTL data from the Framingham Heart Study (FHS) eQTL database[11] accessed through the NCBI Molecular QTL Browser
**Haplotype block around index SNP defined using 1000 Genomes Phase 3 AFR data

Supplementary Table 3: Local replication of established T2D loci in the AADM Study

Chr	Pos	Locus	Population	Index SNP	r2	Best SNP	MAF	BETA	SE	PVAL	df	Adj. P
1	40046093	MACF1	European	rs3768321	0.5641	rs111768751	0.0138	0.536	0.2008	7.59E-03	4.19	3.19E-02
1	154320191	ATP8B2	Japanese	rs67156297	0.3723	1:154320191	0.0135	0.6282	0.208	2.53E-03	8.49	2.14E-02
2	43524295	THADA	European	rs6757251	0.6421	rs534246205	0.2919	-0.1422	0.0512	5.46E-03	8.45	4.62E-02
2	57413142	CCDC85A	Japanese	rs1116357	0.3308	rs56137178	0.2368	0.1727	0.0545	1.54E-03	6.92	1.07E-02
2	161339964	RBMS1	non-European	rs1563575	0.3083	rs62175963	0.2465	0.1669	0.0543	2.11E-03	6.09	1.28E-02
4	185721370	ACSL1	Novel 1000G signal	rs60780116	0.475	rs11936062	0.6677	-0.1493	0.0488	2.24E-03	19.37	4.33E-02
6	32523888	HLA-DQA1	Novel 1000G signal	rs9271774	0.3458	rs66553215	0.6361	0.1939	0.0545	3.73E-04	7.8	2.92E-03
6	126657472	CENPW	European	rs11759026	0.3055	rs4897175	0.3383	0.1634	0.0497	1.01E-03	2.24	2.26E-03
9	22289853	DMRTA1	Japanese	rs1575972	0.9343	rs12000501	0.3117	-0.2159	0.0494	1.22E-05	13.85	1.69E-04
9	84380739	TLE1	European	rs9410573	0.3095	rs7033983	0.9656	0.4575	0.1423	1.31E-03	27.03	3.53E-02
10	12246105	CDC123/ CAMK1D	European	rs11257659	0.5412	rs1320195	0.3439	0.1687	0.0497	6.79E-04	25.76	1.75E-02
10	94281685	HHEX/IDE	European	rs11187140	0.6385	rs10882074	0.0468	-0.399	0.1136	4.46E-04	9.12	4.07E-03
11	2193597	MIR4686	Japanese	rs7107784	0.539	rs10770140	0.456	-0.1793	0.0473	1.48E-04	17.18	2.54E-03
11	17418477	KCNJ11	European	rs5219	0.9123	rs757110	0.9168	-0.2223	0.0621	3.46E-04	10.46	3.62E-03
13	80765272	SPRY2	European	rs11616380	0.3617	rs2876754	0.5826	0.1779	0.0503	4.06E-04	15.56	6.32E-03
18	57766512	MC4R	other	rs1942880	0.7766	rs1539952	0.2322	0.1586	0.0531	2.85E-03	9.78	2.79E-02

Supplementary Table 4: Top hits from meta-analysis of African ancestry – AADM and five-cohort African American - studies

**SNP**	**GENE**	**Weighted N**	**Z score**	**P-value**	**Direction**	**Comments**
rs386418874	TCF7L2	13830	6.536	6.33E-11	++	Known T2D locus
rs4532315	Intergenic	13830	-5.734	9.80E-09	--	No known association with any phenotype;
rs4072825	TH-INS	13830	5.584	2.35E-08	++	Reported T2D GWAS locus[1]; in the IGF-INS-TH region associated with T2D and metabolic phenotypes; associated with MODY and transient neonatal diabetes; Mutations associated with Segawa syndrome (a neurological syndrome)
rs111248619	KCNQ3	13830	5.559	2.71E-08	++	Potassium voltage-gated channel. Associated with neonatal epilepsy. Not previously known to be associated with T2D;

Supplementary Notes

Supplementary Note 1: ZRANB3 primers

EF1alpha
FWD - CTGGAGGCCAGCTCAAACAT
REV - ATCAAGAAGAGTAGTACCGCTAGCATTAC

RPIII
FWD - TCTGGAGGACTGTAAGAGGTATGC
REV - TCTGGAGGACTGTAAGAGGTATGC

ACTIN
FWD - CGAGCTGTCTTCCCATCCA
REV - CGAGCTGTCTTCCCATCCA

ZRANB3
FWD - GAGACATCAACTTGGTGGAGAG
REV - TTGTATCCGCGGAGGTTTG

INS
FWD - TGGTCGATGCCCTTTATCTGG
REV - AGATGCTGCAGGGTTTGTGG

GCK
FWD - TGAAAGTGGGTGAGGATGAAG
REV - CTGCATATCTGACTTCCTGGAC

OPG/TNSF13B
FWD - GGAAGAGCAGGGCACTATTT
REV - GCCGACCACGTTCTTCTTTA

Supplementary References

1 Imamura, M. *et al.* Genome-wide association studies in the Japanese population identify seven novel loci for type 2 diabetes. *Nat Commun* **7**, 10531, doi:10.1038/ncomms10531 (2016).
