# Supplementary material for: ZRANB3 is an African-specific type 2 diabetes locus associated with beta-cell mass and insulin response
Source: Nat Commun. 2019 Jul 19;10:3195. doi: 10.1038/s41467-019-10967-7 (PMC6642147; doi:10.1038/s41467-019-10967-7)
Supplement: Supplementary file 2 — Reporting Summary [file 41467_2019_10967_MOESM2_ESM.pdf]

nature research

Corresponding author(s): Charles N. Rotimi, PhD

Last updated by author(s): May 28, 2019

Reporting Summary

Nature Research wishes to improve the reproducibility of the work that we publish. This form provides structure for consistency and transparency in reporting. For further information on Nature Research policies, see Authors & Referees and the Editorial Policy Checklist.

Statistics

For all statistical analyses, confirm that the following items are present in the figure legend, table legend, main text, or Methods section.

n/a	Confirmed	
☐	☒	The exact sample size (*n*) for each experimental group/condition, given as a discrete number and unit of measurement
☐	☒	A statement on whether measurements were taken from distinct samples or whether the same sample was measured repeatedly
☐	☒	The statistical test(s) used AND whether they are one- or two-sided<br>*Only common tests should be described solely by name; describe more complex techniques in the Methods section.*
☐	☒	A description of all covariates tested
☐	☒	A description of any assumptions or corrections, such as tests of normality and adjustment for multiple comparisons
☐	☒	A full description of the statistical parameters including central tendency (e.g. means) or other basic estimates (e.g. regression coefficient) AND variation (e.g. standard deviation) or associated estimates of uncertainty (e.g. confidence intervals)
☐	☒	For null hypothesis testing, the test statistic (e.g. *F*, *t*, *r*) with confidence intervals, effect sizes, degrees of freedom and *P* value noted<br>*Give P values as exact values whenever suitable.*
☒	☐	For Bayesian analysis, information on the choice of priors and Markov chain Monte Carlo settings
☒	☐	For hierarchical and complex designs, identification of the appropriate level for tests and full reporting of outcomes
☒	☐	Estimates of effect sizes (e.g. Cohen's *d*, Pearson's *r*), indicating how they were calculated

Our web collection on statistics for biologists contains articles on many of the points above.

Software and code

Policy information about availability of computer code

Data collection	No software was used
Data analysis	GMMAT R package<br>GEMMA<br>METAL<br>EUGENE

For manuscripts utilizing custom algorithms or software that are central to the research but not yet described in published literature, software must be made available to editors/reviewers. We strongly encourage code deposition in a community repository (e.g. GitHub). See the Nature Research guidelines for submitting code & software for further information.

Data

Policy information about availability of data

All manuscripts must include a data availability statement. This statement should provide the following information, where applicable:

- Accession codes, unique identifiers, or web links for publicly available datasets
- A list of figures that have associated raw data
- A description of any restrictions on data availability

The GWAS summary statistics are available at dbGap (accession number pending) under terms consistent with the ethical approvals governing the study. The RNA-Seq datasets are accessible online at the Gene Expression Omnibus (GEO) under the accession “GSE125354” [https://www.ncbi.nlm.nih.gov/geo/query/acc.cgi?acc=GSE125354]. All other data are contained within the article and its supplementary information or upon reasonable request from the corresponding author.

Field-specific reporting

Please select the one below that is the best fit for your research. If you are not sure, read the appropriate sections before making your selection.

☒ Life sciences ☐ Behavioural & social sciences ☐ Ecological, evolutionary & environmental sciences

For a reference copy of the document with all sections, see nature.com/documents/nr-reporting-summary-flat.pdf

Life sciences study design

All studies must disclose on these points even when the disclosure is negative.

Sample size	The power of the study for discovery was estimated assuming an alpha of 5 X 10-8. For a variant with a minor allele frequency (MAF) of 0.05, the study has 80% power to detect a genetic risk ratio (GRR) of 1.7 and 94% power to detect a GRR of 1.8. For a variant with MAF of 0.10, the study has 82% power to detect a GRR of 1.5 and 98% power to detect a GRR of 1.6.
Data exclusions	There were no data exclusions for this analysis
Replication	To evaluate replication in another African population, we examined the significant variants in South African Zulu T2D cases and controls from the Durban Diabetes Study (DDS) and the Durban Diabetes Case-Control Study (DCC). A total of 2,578 Zulu participants were included in the replication study
Randomization	Not applicable because this was an observational cross-sectional study, not an intervention study
Blinding	Not applicable because this was an observational case-control study with no intervention and no allocation to groups

Reporting for specific materials, systems and methods

We require information from authors about some types of materials, experimental systems and methods used in many studies. Here, indicate whether each material, system or method listed is relevant to your study. If you are not sure if a list item applies to your research, read the appropriate section before selecting a response.

Materials & experimental systems

n/a	Involved in the study	
☒	☐	Antibodies
☒	☐	Eukaryotic cell lines
☒	☐	Palaeontology
☐	☒	Animals and other organisms
☐	☒	Human research participants
☒	☐	Clinical data

Methods

n/a	Involved in the study	
☒	☐	ChIP-seq
☒	☐	Flow cytometry
☒	☐	MRI-based neuroimaging

Animals and other organisms

Policy information about studies involving animals; ARRIVE guidelines recommended for reporting animal research

Laboratory animals	Zebrafish (Danio rerio), 5 days post fertilization, Tg(insa:mCherry) and Tubingen strain.
Wild animals	The study did not include wild animals
Field-collected samples	The study did not include field-collected samples
Ethics oversight	All experiments performed on animals have been approved by the University of Maryland Baltimore IACUC under protocol approval number 1218020

Note that full information on the approval of the study protocol must also be provided in the manuscript.

Human research participants

Policy information about studies involving human research participants

Population characteristics	The discovery study sample include 5,231 participants from the AADM study participants, including 2,342 type 2 diabetes (T2D) cases and 2,889 controls. T2D was defined by American Diabetes Association (ADA) criteria. Mean age of T2D cases was 55 years and 46 years in controls. Mean BMI was 26.8 kg/m2 in T2D cases and 26.5 kg/m2 in controls. T2D patients had a median fasting glucose of 153 mg/dl (8.5 mmol/L) compared to 85 mg/dl (4.7 mmil/L) in controls.

Recruitment	Participants were Africans enrolled through major medical centers in Nigeria (Ibadan, Lagos and Enugu), Ghana (Accra and Kumasi) and Kenya (Eldoret). Participants identified at each center were first consented for the study and underwent the same enrollment procedures, which included collection of demographic details, medical history and clinical examination. Since the participants were recruited from cities or urban areas, they may not be representative of people living in rural areas.
Ethics oversight	The study protocol was approved by the institutional ethics review board (IRB) of the National Institutes of Health/National Human Genome Research Institute (protocol HG-09-N070) and the IRBs of each participating institution, including University of Lagos, Lagos, Nigeria; College of Medicine, University of Ibadan, Ibadan, Nigeria; University of Nigeria Teaching Hospital, Enugu, Nigeria; University of Science and Technology, Kumasi, Ghana; University of Ghana Medical School, Accra, Ghana.

Note that full information on the approval of the study protocol must also be provided in the manuscript.
